# Strategies for Optimizing Genetic Mouse Models to Enhance the Understanding of Parkinson’s Disease

**DOI:** 10.3390/biomedicines14051162

**Published:** 2026-05-20

**Authors:** Zhiqiang Shen, Linlin Ma, George D. Mellick

**Affiliations:** 1 Institute for Biomedicine and Glycomics, Griffith University, Brisbane, QLD 4111, Australia; zhiqiang.shen@griffithuni.edu.au; 2 School of Environment and Science, Griffith University, Brisbane, QLD 4111, Australia

**Keywords:** Parkinson’s disease, mouse models, genetic models, gene-environment interactions, non-motor symptoms

## Abstract

**Background**: Parkinson’s disease (PD) has become the fastest-growing neurodegenerative disorder worldwide. A valuable approach for unraveling the disease’s mechanisms and new therapeutic targets involves investigating the PD-causing genes identified in families exhibiting the Mendelian inheritance of parkinsonism. **Methods**: In this article, we review how genetically modified mouse models can be employed to decipher the genetic architecture of PD. **Results**: We first discuss how well the human motor and non-motor symptoms of PD are currently evaluated in these PD mouse models, highlighting limitations. The pathogenic roles of five inherited PARK genes in PD are then extensively examined through their respective genetic mouse models in terms of phenotypic and cellular impacts. Furthermore, we discuss the strengths and weaknesses of existing transgenic mouse models and highlight significant accomplishments and advancements in this field from 2018 to the present. **Conclusions**: Building upon the current understanding of PD, we propose potential directions for enhancing genetic mouse models to further unveil the underlying mechanisms of PD and advance therapeutic research.

## 1. Introduction

Parkinson’s disease (PD) is a slowly progressive, devastating neurodegenerative disorder with two hallmark neuropathological features: intracellular protein α-synuclein aggregation, called Lewy pathology, in several different brain regions, and prominent progressive death of dopaminergic neurons in the substantia nigra pars compacta (SNpc) [1,2,3]. According to human clinical autopsy data, Braak concluded that the propagation of α-synuclein (Lewy pathology) commences in the vagal nerve and lower brainstem, including the anterior olfactory nucleus, and peripheral and central medullary autonomic neurons, before progressing through the midbrain to eventually reach the neocortex [4,5]. This hypothesis has been successfully replicated in mouse models, where targeted α-synuclein seeding in the gut or olfactory bulb induces progressive pathology in line with Braak staging [6]. Notably, neuronal degeneration in PD is most pronounced in the ventrolateral tier of the SNpc, particularly in early disease stages [7,8]. Evidence suggests that degeneration of dopaminergic neurons begins at the dopaminergic terminals and gradually regresses toward the soma [9]. Postmortem tissue analysis and in vivo studies further support the spatial and temporal relationship between α-synuclein pathology and neurodegeneration [10,11,12].

The clinical hallmarks of PD are broadly categorized into motor symptoms and non-motor symptoms. Dopamine deficiency is the primary driver of the core motor symptoms, while non-motor manifestations are heavily driven by noradrenergic, serotonergic, and cholinergic degeneration in PD patients [7,13]. The onset of motor symptoms is preceded by a prodromal phase, lasting many years, characterized by non-motor symptoms, including idiopathic rapid eye movement sleep disorder, depression, excessive daytime somnolence, fatigue, hyposmia, anxiety, constipation, hypotension, urinary dysfunction, and autonomic dysfunction [3,7,13,14,15,16,17,18]. During the pre-motor period, neuronal loss in the SN is relatively limited [10]. Many additional non-motor symptoms experienced by PD patients are non-specific and thus supportive but not diagnostic. Some of these include, gait disturbance, dystonia, gastrointestinal (GI) dysfunction, depression, anxiety, and cognitive changes. Nonetheless, these symptoms offer valuable indicators for PD modeling in animals [1,2,7,13,19,20,21,22,23,24,25]. The presence of parkinsonism remains a prerequisite for PD diagnosis. However, motor symptoms only emerge after a critical threshold of dopaminergic neuron loss is reached [9,10]. The onset of motor symptoms, such as bradykinesia, resting tremor, rigidity, akinesia, axial deformities, dysphagia, postural instability, and gait disorder, form the primary requirement for clinical diagnosis [1,2,19,20,21,22,26]. The progressive nature of PD highlights the importance of understanding the spatiotemporal dynamics of neurodegeneration and α-synuclein pathology. Such diverse manifestations result from multifactorial mechanisms associated with PD at the cellular level, including apoptosis, mitochondrial dysfunction, oxidative stress, synaptic dysfunction, deficiency in the lysosome and proteasome system, axonal transport dysfunction, calcium homeostasis disorder and neuroinflammation [2,3].

Animal models, including drosophila, zebrafish, primates, and rodents, have been employed to investigate various aspects of PD for research purposes [27,28,29,30]. Rodents, such as mice, are particularly advantageous due to their ease of breeding, short life cycles, and ability to be genetically modified. Moreover, the developmental processes of the rodent brain closely resemble those of humans, making rodents an excellent model for studying genes related to PD [31]. Rodent models accounted for 85% of all research articles on animal studies of PD from 1990 to 2018 [28,32]. Many transgenic mouse models have been developed attempting to reproduce specific pathological features and motor symptoms of PD observed in humans.

However, it should be noted that mice do not naturally develop the full clinicopathological spectrum of PD. While mice can be induced to exhibit certain parkinsonian symptoms, the way these symptoms manifest may vary between species [33,34]. Developing genetic mouse models that fully replicate PD is challenging due to the inherent differences between mice and humans and the complex clinical presentation of the disease. Most transgenic mouse models focus on a limited number of well-established genes linked to inherited PD. A comprehensive review conducted by Breger and Fuzzati-Armentero [35] provided an extensive overview of transgenic mouse models of PD up to mid-2018. These authors emphasize the need to broaden the characterization of these models beyond the assessment of neurodegeneration and motor impairments. Most current models cannot reproduce the chronic progressive nature of human PD. The models do not adequately reflect the prodromal phase or slow evolution seen in patients. Furthermore, complete recapitulation of selective vulnerability, particularly the tier-specific loss in the SNpc, and the complex interaction between α-synuclein pathology, neuroinflammation, and systemic factors are often lacking. Inconsistent expression levels, promoter effects, and variability in protocols also contribute to differences in outcomes across studies.

Since 2018, over 600 primary publications have explored transgenic mouse models of PD, offering additional insights into their utility for deciphering the genetic foundations of the human condition. However, the rapid expansion of methodological diversity and model heterogeneity has generated a fragmented body of evidence. In this review, we aim to provide a comprehensive strategic roadmap for future research that not only consolidates existing findings but also critically assesses their translational relevance and their value for understanding the human disease. Specifically, we aim to:

1. Systematically review, categorize, and summarize recent literature on transgenic PD mouse models, with a particular focus on novel phenotypic and pathological characteristics, and provide a critical evaluation of the strengths and limitations of these model-specific characteristics.
2. Explore the multifactorial model systems that incorporate both genetic susceptibility and environmental triggers, particularly the toxin–gene interaction and polygenic models which may better recapitulate the multifactorial and progressive nature of PD; and
3. Summarize and update the application and interpretation of behavioral tests tailored to PD symptoms, proposing standardized criteria for classifying results to facilitate meaningful comparisons across different models.

Furthermore, this review aims to critically evaluate how transgenic mouse studies can be further refined to enhance their translational relevance, ultimately advancing our understanding of the genetic contributions to PD pathogenesis.

## 2. Methods

We conducted an extensive literature search on PubMed, using the following search keywords: (gene name) AND (mouse model) AND (Parkinson’s disease). To date, transgenic mouse models have been developed for 13 PARK genes (summarized in Table 1 and Table 2). This review focuses on the five most extensively studied genes in the family of PARK loci, namely, *SNCA*, *PRKN*, *LRRK2*, *PINK1*, and *DJ-1*, each of which has established genetic mouse models and well-characterized pathogenetic roles in PD. While Table 2 summarizes the existing transgenic mouse models for the remaining eight genes, these are not discussed in depth due to the limited validation and paucity of available information for these models. *SNCA* and *LRRK2*-linked PD are autosomal dominant, while *PRKN*, *PINK1* and *DJ-1*-linked PD are autosomal recessive. The following sections provide a detailed overview of their genetic architecture, molecular mechanisms, applications in mouse models, and latest research developments, offering a comprehensive view on the genetic contributions to PD. An introduction to the main genes, their pathogenic mutations and mutation effects are summarized (Table 1).

Although less frequently studied, transgenic mouse models targeting the other eight PARK genes (PARK 5, 9, 11, 13, 14, 17, 19, and 20) have also been recognized as pivotal tools for unraveling the pathogenesis of PD. Sixteen transgenic mouse models based on these eight genes are summarized in Table 2, selected according to the following criteria: (1) direct impact on viability and development, (2) evidence of PD-related neurodegeneration and synucleinopathy, (3) manifestation of motor and/or non-motor PD-like deficits, and (4) utility in mechanistic exploration of PD pathogenesis.

## 3. Clarifying Model Capability

Monogenic forms of PD follow Mendelian inheritance patterns and are classified as either autosomal dominant or autosomal recessive. Accordingly, transgenic mouse models of PD are broadly categorized into knockout and overexpression systems, each tailored to dissect distinct genetic mechanisms. Knockout models, designed to mimic autosomal recessive PD, eliminate or impair gene function to investigate proteostatic failure in dopaminergic neurons. Conversely, overexpression models replicate dominant gain-of-function pathologies, enabling studies of α-synuclein aggregation dynamics and neurotoxicity. These genetic models are further classified into germline and conditional modifications. Germline models involve global gene alterations affecting all cells in the animal, whereas conditional models use tissue-specific promoters or inducible systems to restrict gene expression changes to particular regions or time points. This flexibility allows researchers to explore the gene’s effects in specific biological contexts, providing more targeted insights into the underlying mechanism.

There has been significant progress in genetic research related to PD. Various potential PD-related genes with numerous pathogenic mutations have been identified across diverse populations and lineages. An overview of PD-related genetic pathways and associated animal models has been comprehensively summarized elsewhere [35,101]. Based on this foundation, this section focuses on five monogenic forms of PD that have been extensively characterized in the literature. We summarize their established phenotypic and pathological features, together with recent advances, to illustrate how genetic mouse models have enhanced our understanding of this highly complex neurodegenerative disorder (Table 3).

### 3.1. SNCA (PARK1/4)

#### 3.1.1. Physiological Functions of α-Synuclein

The human *SNCA* gene (Gene ID: 6622, National Library of Medicine), located on chromosome 4q22.1, encodes the α-synuclein protein composed of 140 amino acids [113]. In healthy brains, α-synuclein is predominantly expressed in presynaptic terminals in a monomeric, unstructured form [114,115,116,117,118]. It acts as a non-classical chaperone protein that binds to soluble N-ethylmaleimide sensitive factor attachment proteins receptor (SNARE) protein synaptobrevin-2 and contributes to the assembly of SNARE-complex to facilitate synaptic vesicle trafficking and neurotransmitter release at presynaptic nerve terminals [119]. Mice lacking α-synuclein are viable and fertile but do not exhibit any noticeable pathological abnormalities or motor deficits [120,121]. Furthermore, while individual knockouts of α-synuclein or β-synuclein do not impact dopamine levels, double knockout leads to a decrease in dopamine levels, suggesting functional redundancy between the two synucleins, likely due to their molecular similarity [122]. While the knockout models provide valuable insights into the physiological function of α-synuclein, it is important to note that all pathogenic *SNCA* mutations identified in familial PD are gain-of-function, emphasizing the importance of overexpression or knock-in models for accurately studying disease mechanisms [123].

#### 3.1.2. *SNCA* Mono-Transgenic Mouse Models

Under pathological conditions, toxic oligomers and fibrillar aggregates of α-synuclein induce selective and progressive neuronal death by disrupting mitochondrial function, impairing lysosomal activity, and altering calcium homeostasis [124]. Accordingly, transgenic mice overexpressing wild-type (WT) α-synuclein or carrying *SNCA* mutations (e.g., *SNCA*^p.A53T^, *SNCA*^p.E46K^, *SNCA*^p.A30P^, see Table 1) have been widely employed to replicate PD symptoms, including olfactory and autonomic dysfunction, cognitive deficits, circadian rhythm disturbances, and early motor deficits, along with the progressive formation of α-synuclein-positive inclusions [35,123,125,126,127,128].

Despite all the advances achieved through these α-synuclein models, a persistent challenge remains: the inconsistent and often limited nigrostriatal neurodegeneration observed across models. As highlighted in previous reviews, most mouse *SNCA* transgenic models do not exhibit dopaminergic neurodegeneration in the nigrostriatal area [127,129,130]. However, there are noteworthy transgenic models with stable nigrostriatal neuron loss. For example, Chesselet and colleagues characterized a model in which full-length, wild-type human α-synuclein was overexpressed under the Thy-1 promoter in C57BL6/DBA2 mice [131]. This model displayed 40% loss of striatal dopamine at 14 months of age, along with olfactory deficits, colonic deficits and progressive motor impairments (as assessed by beam walk and pole tests) [131]. Wakamatsu and colleagues generated a novel mouse model expressing truncated human α-synuclein (residues 1 to 130) on the C57BL6J/B6C3F1 background [132]. Immunoblot analysis revealed approximately 45% dopaminergic neurodegeneration in the SN at two months of age, but no further progressive degeneration was observed up to thirteen months [132]. Similarly, these models highlight the ongoing need for long-term screening, optimization of mouse strains, and refinement of transgenic vectors to more faithfully model the progressive neurodegeneration observed in human PD.

#### 3.1.3. New Developments in α-Synuclein Transgenic Models

The long-term screening and optimization of mouse strains and transgenic vectors are required to refine and improve these *SNCA* transgenic mouse models. Recent studies using the Jackson Laboratory *SNCA* overexpression mouse strain (Stock No. 023837) revealed progressive dopaminergic neurodegeneration, with ~50% loss of tyrosine hydroxylase (TH) positive neurons in the SN and striatum between 4 and 14 months [133]. In addition to brain pathology, aggregation of α-synuclein was observed in peripheral tissues. In B6;C3-Tg(Prnp-SNCA*A53T)83Vle/J mice expressing *SNCA*^p.A53T^, α-synuclein aggregates were found in retina, particularly in the outer plexiform layer, leading to the degeneration of synaptic ribbons, and TH^+^ retinal neurons by 18 months of age [134]. In addition, Lewy body-like aggregates were observed in the colon at 4, 8, and 14 months of age in transgenic mice expressing *SNCA* on a *Snca* knockout background (B6.Cg-Tg(SNCA)OVX37Rwm *Snca^tm1Rosl^*/J) [133]. Meanwhile, enteric and vagus nerve α-synuclein deposition developed in 12-month-old C57BL/6J mice carrying a CRISPR/Cas9 generated *Snca*^G51D^ knock-in mutation and progressed with age [135].

In addition to replicating key neuropathological features of PD, a range of non-motor symptoms were successfully discovered in *SNCA* transgenic mouse models, such as gastrointestinal dysfunction, olfactory deficits, anxiety and sleep disorders, as well as associated cellular-level pathologies. For example, colonic motility deficits have been observed in double-PAC-transgenic mice expressing *SNCA*^p.A53T^ using the bead expulsion test and whole-gut transit time test, on a mixed genetic background of 129S6/SvEvTac × FVB/N [136]. Injection of the *SNCA* gene into the hippocampus of 8-week-old C57BL/6 J mice using an AAV vector resulted in anxiety-like behaviors in behavioral tests, including the tail suspension test, forced swimming test, elevated cross maze, and open field test [137]. Taguchi and colleagues reported a progressive sleep disorder phenotype in BAC transgenic C57BL/6J mice expressing *SNCA*^p.A53T/−^, characterized by increased electromyography (EMG) variance between 5 and 13 months of age [106]. Notably, pre-motor olfactory deficits emerged by 6 months of age in B6.Cg-*2310039L15Rik*^Tg(Prnp-SNCA*A53T)23Mkle^/J mice expressing *SNCA*^p.A53T^, closely resembling the prodromal phase of human PD [107]. In BAC-h*SNCA*^WT^ overexpression mice at thirteen months old on a C57BL/6J background, compromised blood–brain barrier and degeneration of striatal blood vessels were observed [138]. Compared with WT mice, expression of *SNCA*^p.A53T^ in carriers (FVB;129S6-Tg(SNCA*A53T)1Nbm *Snca^tm1Nbm^* Tg(SNCA*A53T)2Nbm/J, Jax strain number: 010799) exhibited a significantly increased proportion of abnormal red blood cells [139]. The expression of *SNCA*^p.A53T^ in mice (B6;C3-Tg (Prnp-SNCA*A53T)83Vle/J) also induced the lymphadenectasis, enlargement of the lymph sinus, and upregulation of inflammatory cytokines (such as IL-1β, IL-6 and TNF-α) [140]. Supporting these findings, post-mortem examination of PD patients’ brains showed increased string vessel formation, consistent with microvascular degeneration in PD [141]. Collectively, these findings underscore the multifaceted role of α-synuclein in both central and peripheral pathologies in PD, emphasizing the relevance of *SNCA* transgenic mouse models for capturing these broader aspects of the disease.

#### 3.1.4. Mechanistic Insights and Therapeutic Implications

*SNCA* transgenic models, while imperfect, have provided valuable insights into the mechanistic links between α-synuclein pathology, microglia activation and PD progression. Microglial activation through pathways involving NOD-, LRR- and pyrin domain-containing protein 3 inflammasomes, interleukin-1 receptor, and fractalkine signaling exacerbates neurodegeneration in PD models [142,143,144,145]. Another highlight is the interaction between α-synuclein and mitochondrial dysfunction and oxidative stress [146]. Disruption of mitochondrial transmembrane potential caused by α-synuclein-induced complex I inhibition leads to increased oxidative stress and mitophagy [116]. In turn, increased levels of oxidative stress can further accelerate the aggregation of α-synuclein, creating a self-reinforcing pathogenic loop [147].

The recognition of α-synuclein’s pivotal role in PD pathogenesis has driven substantial progress in therapeutic innovation. Recent efforts to enhance translational validity in preclinical studies include the use of refined animal models that better replicate the spatiotemporal progression of human PD. These models are increasingly used to evaluate compounds that inhibit α-synuclein aggregation, block its cell-to-cell transmission, or promote its clearance through immunotherapy or autophagy enhancement (Table 4).

As shown in Table 4, BIIB054 (Cinpanemab, high affinity scavenger of α-synuclein) and Posiphen (ANVS101/Buntanetap, inhibits α-synuclein expression) represent two different chapters in the effort to develop disease-modifying therapies for PD. BIIB054 is a human-derived monoclonal antibody designed to bind to the N-terminus of extracellular α-synuclein, preventing its “prion-like” spread from one neuron to another. Unfortunately, the development of BIIB054 was terminated following the Phase II clinical trial [158]. The trial results showed that patients treated with BIIB054 did not differ from those receiving placebo in MDS-UPDRS subscale scores or DaT-SPECT scans over a 52-week period [158]. In contrast, Posiphen, a small molecule that inhibits the translation of α-synuclein, has recently completed a Phase III clinical trial (NCT05357989, 2024) by Annovis Bio Inc. The results report significant improvements in both motor and cognitive scales in early PD patients, potentially offering a multi-protein translational approach to neuroprotection [159]. A new phase II and III clinical trial (NCT07284784, 2026) is currently recruiting to examine the long-term safety of Posiphen in participants with PD [160]. These advancements underscore the therapeutic potential of targeting α-synuclein-driven mechanisms and highlight the continued importance of *SNCA* transgenic models in the preclinical evaluation of PD interventions.

### 3.2. LRRK2 (PARK8)

#### 3.2.1. The Role of LRRK2 in PD

The human *LRRK2* gene (NLM, gene ID 120892), located on chromosome 12q12 and comprising 53 exons, encodes a protein of 2527 amino acids called LRRK2. This multidomain protein functions as a kinase and directly phosphorylates threonine/serine residues of several Rab GTPase proteins, such as Rab 1, 5, 7, 8, 10, 12, and 39 [161,162]. Rab GTPases play diverse roles in coordinating intracellular membrane trafficking in cells [163], implicating LRRK2 in various pathogenic pathways associated with PD. The *LRRK2*^p.G2019S^ mutation has been found in approximately 4% of all hereditary PD cases and 1% of sporadic PD cases worldwide, while *LRRK2*^p.R1441C^ has been reported to exhibit over 90% penetrance by age 75 [164,165]. Postmortem studies reveal heterogeneity in *LRRK2*-linked PD, with some patients exhibiting both nigrostriatal neurodegeneration and Lewy pathology, while others showing selective SN degeneration without central Lewy bodies [166,167,168]. Importantly, heterozygous *LRRK2* loss-of-function carriers do not develop PD [169], whereas gain-of-function mutations are associated with elevated PD risk [170]. This dichotomy underpins therapeutic strategies targeting LRRK2 inhibitors to counteract its pathogenic overactivity [171,172,173].

#### 3.2.2. Challenges in Developing *LRRK2* Mono-Transgenic Mouse Models

Despite extensive efforts, developing robust *LRRK2* transgenic mouse models that replicate PD pathology has proven difficult. Overexpression of wild-type or mutant human or mouse LRRK2, regardless of the promoter used, fails to produce consistent neurological or pathological symptoms [174,175,176,177,178,179,180]. Specifically, C57BL/6-based mouse models have not reliably recapitulated any PD pathological hallmarks [174,175,177,181,182,183,184,185]. For example, CMV-driven h*LRRK2*^p.G2019S^ expression in C57BL/6J mice induced significant but modest neurodegeneration (14%) after 19 months, and h*LRRK2*^p.R1441C^ expression on the same background failed to cause significant neurodegeneration in the SN area [102].

The FVB/N mouse strain, widely used due to its high transgenic efficiency, serves as one potential choice for developing *LRRK2* transgenic mouse models. However, it similarly exhibits inconsistent outcomes with respect to the development of a neurodegenerative phenotype. While some studies reported significant loss of TH^+^ cells in the SN of 16-months-old FVB/N mice expressing *LRRK2*^p.R1441C^ [179], other mice (FVB/N-Tg(LRRK2*R1441C)135Cjli/J) observed no such effect [186]. Mice expressing *LRRK2*^p.R1441G^ only exhibited TH^+^ dendrites degenerating in SN pars reticulata without dopaminergic neurodegeneration in SNpc and ventral tegmental area at 10 months [187]. Additionally, FVB/N mice display chaotic circadian rhythm patterns and visual impairment, which may contribute to the inconsistent behavioral test results [188]. These factors, coupled with the inconsistent reproducibility of dopaminergic neurodegeneration undermine the reliability of FVB/N strain for modeling PD pathology.

#### 3.2.3. Reproduction of Neuropathological, Functional and Molecular Features

Although *LRRK2* mutations lead to minimal neurodegeneration in the substantia nigra, they still influence the function and morphology of neurons in C57BL/6 mice [189,190,191]. For example, *Lrrk2*^p.R1441C^ and *LRRK2*^p.G2019S^ mutations contribute to the loss of primary cilia in choline acetyltransferase interneurons of the dorsal striatum [192,193]. Moreover, mutation in *LRRK2*^p.G2019S^ increases nicotinamide adenine dinucleotide phosphate levels, resulting in metabolic changes in neurons [194]. Besides functional changes, *LRRK2* causal variants also promote α-synuclein aggregation. For instance, recent exploration in *LRRK2* transgenic mouse models revealed that the *Lrrk2*^p.R1441G^ variant increased the levels of α-synuclein oligomers in the cortex (30.7%) and striatum (53.2%) by 18 months of age in C57BL/6N mice [103]. These findings suggest that *LRRK2* mutations impact both neuronal function and α-synuclein pathology, confirming its involvement in PD pathogenesis.

These functional changes in neurons and α-synuclein aggregation are further reflected in motor deficits and non-motor manifestations. The expression of *LRRK2*^p.R1441C^ in the C57BL/6J strain caused the progressive impairment of locomotor activity [102]. Similar motor deficits were detected in two mouse strains with expression of *LRRK2*^p.G2019S^ (C57BL/6J-Tg(LRRK2*G2019S)2AMjff/J and B6;C3-Tg(PDGFB-LRRK2*G2019S)340Djmo/J) about 65 weeks old [195,196]. Additionally, expression of mutant *Lrrk2*^p.G2019S^ contributes to skeletal muscle EMG spontaneous potential impairment and reduction in muscle strength and mass [197]. Non-motor phenotypes have also been reported in *Lrrk2*^p.G2019S^/*LRRK2*^p.G2019S^ mouse models. For example, sleep pattern disturbances have been observed in *Lrrk2*^p.G2019S^ knock-in mice, while depression-like behavior and alterations in gut microbiota composition have been described in the FVB/N-Tg(LRRK2*G2019S)1Cjli/J transgenic line expressing *LRRK2*^p.G2019S^ [110,111,198]. Compared with WT mice, *Lrrk2* KO mice showed impaired olfactory discrimination, as they did not significantly increase sniffing time toward the novel odorant (eugenol) relative to the habituation trial [108]. The newly discovered variant, *Lrrk2*^p.R1628P^ was reported to induce intestinal dysfunction in C57BL/6J mice, further expanding the phenotypic spectrum [109].

On the molecular level, *LRRK2* mutations are implicated in PD-related molecular pathways involving inflammation, mitochondrial dysfunction, neurotransmitter transmission and ER stress. For example, transgenic mice expressing *Lrrk2*^p.G2019S^ show increased levels of pro-inflammatory cytokines in the skeletal muscles [197]. Similarly, in the *Lrrk2*^p.G2019S^ knock-in model, this mutation exacerbates mitochondrial DNA damage in the ventral midbrain [199]. Altered transcription resulting from *LRRK2*^p.G2019S^ leads to microglia sensitization and increased inflammation levels across multiple models, including the FVB/N-Tg(LRRK2*G2019S)1Cjli/J and the C57BL/6J-Tg(LRRK2*G2019S)2AMjff/J transgenic mice expressing *LRRK2*^p.G2019S^, as well as C57BL/6J *Lrrk2*^p.G2019S^ knock-in mice and adenoviral vector-mediated expression systems [200,201,202,203,204]. In addition, *LRRK2*^p.G2019S^ has been found to affect glutamatergic synaptic transmission, thereby influencing striatal synaptic plasticity and cognitive learning under stress [205,206,207,208]. These effects have been observed in C57BL/6J-Tg(LRRK2*G2019S)2AMjff/J transgenic mice expressing *LRRK2*^p.G2019S^, as well as in C57BL/6N *Lrrk2*^p.G2019S^ knock-in mice carrying the endogenous mutation. The *Lrrk2*^p.R1441G^ mutation in the C57BL/6N mice leads to decreased synaptogyrin-3 expression, which may impair dopamine reuptake [209]. Furthermore, expression of *LRRK2*^p.G2019S^ in FVB/N mice leads to the accumulation of misfolded proteins in neurons and induces ER stress by promoting the expression of thrombospondin-1/transforming growth factor beta1 [210].

Proteomics analyses in mice have revealed that *LRRK2* mutation leads to alterations in lysosomal proteases, cytoskeletal proteins, and protein translational machinery [211]. In C57BL/6N Tac mice, the increased LRRK2 kinase activity caused by *Lrrk2*^p.G2019S^ knock-in can mistakenly recruit and activate the motor adaptor JNK-interacting protein 4, leading to deficits in autophagosome transport [212]. Additionally, expression of *Lrrk2*^p.G2019S^ gene impairs glutamate clearance by affecting excitatory amino acid transporter 2, potentially leading to glutamate overload and subsequent neurodegeneration in C57BL/6J mice [213]. These insights into proteomic and cellular dysfunction highlight the multifaceted contributions of *LRRK2* mutations to PD pathogenesis and underscore the relevance of LRRK2 as a potential therapeutic target. Furthermore, these findings support the contention that *LRRK2* transgenic mouse models have served as useful tools for studying PD more broadly.

### 3.3. PRKN (PARK2)

#### 3.3.1. The Role of Parkin in PD

The human *PRKN* gene (PARK2) (NLM, gene ID 5071), located on chromosome 6q26 and comprising 13 exons, encodes a protein of 465 amino acids called Parkin, which acts as an E3 ubiquitin-protein ligase [214]. In the ubiquitin system, ubiquitin-protein ligase mediates the transfer of ubiquitin from the ubiquitin-conjugating enzyme to the substrate protein. The ubiquitin-tagged protein is then targeted for intracellular degradation [215]. When mitochondrial depolarization occurs, PINK1-dependent phosphorylation of Parkin Ser 65 is essential for the formation of Parkin ubiquitin-ester intermediates [216]. This phosphorylation enables conformational changes in the RING0 domain, exposing Parkin’s catalytic core only after ubiquitin itself is also phosphorylated at Ser65 [217]. Phosphorylated polyubiquitin chains on the mitochondrial outer membrane (MOM), generated by PINK1, then serve as a signal for Parkin translocation [218,219]. Once recruited, activated Parkin mediates the ubiquitylation of several proteins on MOM, including TOMM70A, HK1 and MFN1 for mitophagy [218,219,220,221,222]. In addition to its role in mitochondrial quality control, Parkin is also involved in the cell rescue signaling pathway and mitochondrial biogenesis via peroxisome proliferator-activated receptor gamma coactivator 1-alpha [223]. Therefore, Parkin plays an essential role in eliminating dysfunctional mitochondria [224,225,226].

#### 3.3.2. Neurological Deficits in *PRKN*-Based PD Mouse Models

Over 200 PD-associated *PRKN* loss-of-function mutations have been identified. Expression of *PRKN*^p.Q311Ter^ leads to the accumulation of glutamate kainate receptors, contributing to neurodegeneration in the SN of C57BL/6N mice at 6 months of age [227]. Synaptotagmin-11, a physiological substrate of Parkin, has also been implicated to play an essential role in Parkin deficiency-induced neurotoxicity [228]. In *Prkn* exon 2 knockout C57BL/6J mice, synaptotagmin-11 accumulates and its overexpression induces the loss of TH^+^ neurons in the SNpc along with abnormal behaviors in methamphetamine-induced rotational test and gait analysis [228].

Beyond neuron death, deficiency affects key neuronal processes. In *Prkn* exon 3 knockout C57BL/6 mice, the over-acetylation of the microtubule system in nigrostriatal neurons appears to contribute to mitochondrial damage via disorientating the transport of mitochondria and subsequent axonal degeneration [229]. Compared with control cells, the lack of normal Parkin leads to lower complexity of human iPSC-derived neurons, including shorter neurite length, fewer terminal number, and fewer branch points [230]. In a recent study, Regoni and colleagues used high magnification fluorescent microscopy (100x) to observe significantly more cytoplasmic vacuolization and disruptions in mitochondrial ultrastructure in the SN dopaminergic neurons of *Prkn*^p.R275W^ knock-in C57BL/6N Tac mice at just one month of age, confirming the essential role of Parkin in early neuronal development and mitochondrial maintenance [105]. Apart from reproducing dopaminergic neurodegeneration, newer *Prkn* mutation mouse models, such as *Prkn*^p.S65A^ C57BL/6J and *Prkn*^p.R275W^ C57BL/6N Tac, have been reported to exhibit marked motor deficits and balance impairment in mice [104,105].

#### 3.3.3. The Strain-Dependent Phenotypic Differences

However, traditional *Prkn* knockout models targeting exons 2, 3, or 7 have failed to consistently recapitulate key phenotypic changes related to human PD [231,232,233,234,235,236]. In contrast, BAC- *PRKN*^p.Q311Ter^ FVB mice, which carry a nonsense mutation in exon 8 (*PRKN*^p.Q311Ter^) [52], exhibit significant dopaminergic neurodegeneration in the substantia nigra and striatum with motor deficits at 16 months of age [237]. These mice also exhibited progressive accumulation of α-synuclein in the SN (at 16 months) [237] and significant neuroinflammation (at 12 months) and motor deficits (>6 months) [238]. These findings suggest that the impact of *PRKN* mutations is highly strain-dependent and models based on the C57BL/6 background may have limited capacity to faithfully reproduce key PD pathology and phenotypes.

### 3.4. PINK1 (PARK6)

#### 3.4.1. The Role of PINK1 in Mitochondrial Function and PD

The human *PINK1* or PARK6 gene (NLM, gene ID 65018), located on chromosome 1p36.12, encodes a 581-amino acid serine/threonine kinase PINK1 [55]. PINK1 plays a key role in mitochondrial quality control through its involvement in the ubiquitin-proteasome system. The N-terminal of the PINK1 precursor protein contains mitochondrial targeting sequences [239], allowing its import into mitochondria, where it anchors via interactions with proteins synaptojanin 2a and synaptojanin 2 binding protein [240]. Under normal conditions, mitochondrial proteases such as presenilin-associated rhomboid-like protease and mitochondrial processing peptidase mediate the import, processing, and degradation of the PINK1 precursor [241,242,243]. When mitochondrial depolarization occurs, PINK1 accumulates on the MOM and phosphorylates ubiquitin and Parkin to initiate mitophagy [216,217,219,241,242]. Furthermore, it has been shown that PINK1 binds to the pro-autophagic protein Beclin1 to enhance autophagy [57]. Loss-of-function mutations in *PINK1* are associated with autosomal recessive early-onset PD and impair its role in mitochondrial homeostasis and autophagic regulation [55,57,60,61].

#### 3.4.2. The Limitations and the Developments in *PINK1* Transgenic Mouse Models

Similar to *Prkn* knockout models, *Pink1*-deficient mice carrying deletion of exons 2–5 on a C57BL/6 × 129/Sv background or exons 4–7 on a C57BL/6J × 129/SvEv^Brd^ background do not show dopaminergic neurodegeneration [244,245]. Although a decrease in dopamine levels and inflammatory cytokines were detected in exon 4–5 knockout-induced *Pink1*-deficient C57BL/6 × 129/Sv hybrid mice, no TH^+^ neurodegeneration was observed [246]. Filograna and colleagues also reported no PD-like phenotype in *Pink1* knockout C57BL/6N mice [247]. Transgenic models expressing the *Pink1*^p.G309D^ mutation showed α-synuclein aggregation in the midbrain without loss of TH^+^ neurons in the SN in 129/SvEv mice [248].

The development and investigation of *PINK1* mouse models are ongoing. Recent studies suggest that *PINK1*-related phenotypes may be more nuanced and develop with age. Despite the initial lack of an identified motor phenotype, more recent re-examinations revealed non-motor symptoms such as depression and anxiety in aging *Pink1*-deficient mice on C57BL/6 × 129/Sv and C57BL/6J (129S4/SvJae) background [112,249]. Moreover, age-dependent changes in primary cilia morphology have been observed in striatal and cholinergic neurons of *Pink1* exons 2 and 3 knockout C57BL/6 J mice, similar to the observations in *Lrrk2*^p.R1441C^ and *LRRK2*^p.G2019S^ mouse models, suggesting shared pathogenic pathways [192,193,250]. These findings highlight the emerging role of PINK1 in PD pathogenesis and emphasize the need for further refinement of *PINK1* transgenic mouse models to more faithfully replicate both motor and non-motor aspects of the disease.

### 3.5. DJ-1 (PARK7)

#### 3.5.1. The Role of DJ-1 in Oxidative Stress and PD

The human *DJ-1* gene (NLM, gene ID 11315), located on chromosome 1p36.23, encodes a 189-amino acid protein. Homodimeric DJ-1 is present in the cytoplasm, mitochondria, and nucleus. It functions as an oxidative stress sensor, contributing to the elimination of reactive oxygen species such as hydrogen peroxide [251,252]. This suggests that DJ-1 plays a neuroprotective role in the brain [253]. Pathogenic mutations in *DJ-1* are typically loss-of-function mutations associated with autosomal recessive early-onset parkinsonism [254], implicating DJ-1 in the pathogenic mechanism of PD [64,65,66,67,68,69].

#### 3.5.2. Behavioral and Cellular Changes in *DJ-1* Mono-Transgenic Mouse Models

The existing *DJ-1* mono-transgenic mouse models poorly recapitulate notable parkinsonian phenotypic features [255,256,257,258,259,260,261]. However, some subtle changes have been reported in these animals. While no changes in the substantia nigra were observed, noticeable TH^+^ neurodegeneration was found in the retinas of *Dj-1* exon 2 knockout C57BL6/J mice, leading to increased light sensitivity of the eyes [256,261]. Although typical neuropathological features cannot be reproduced in mouse models, behavioral phenotypes have emerged in recent studies. For example, in *Dj-1* exon 2 knockout B6/129 mice, reduced locomotor activity was observed in open field test at an age of three months [262]. Similarly, on a C57BL/6J × 129/SvJ background, knockout of *Dj-1* exon 2 resulted in gait abnormalities and a decline in grip strength at 16 months of age [257]. In a separate model on a C57BL/6 background, the knockout of *Dj-1* exons 3–5 led to motor deficits (at 10 months) [263]. These findings suggest that DJ-1 plays a role in motor function and oxidative stress regulation, although its dopaminergic impact in current mouse models appears modest.

Beyond behavioral alterations, *DJ-1* mouse models have provided insights into underlying mechanisms of PD, particularly inflammation and mitochondrial function. Excessive activation of the apoptosis-related p53 pathway was reported and contributed to apoptosis and inflammation in the colon after *Dj-1* knockout in C57BL/6J mice [264]. Increased microglial activation caused by the nuclear factor kappa B pathway was observed in *Dj-1* exon 2 knockout mice on a B6/129 background [265]. In heterozygous mutant mice on a C57B/6 × 129 mixed background, *Dj-1* deficit (exons 1–5 knockout) attenuated the proliferation of astrocytes and monocyte infiltration, and delayed the recovery from brain injury, further contributing to PD [266]. Through proteome analysis and further research, Ozawa found that the DJ-1 protein is essential for the nitrosylation of Parkin, which is important for maintaining mitochondrial function, using *Dj-1* exon 2 knockout mice on a C57BL/6 × 129 hybrid background [267]. These findings suggest that DJ-1 plays a critical role in regulating inflammatory responses and mitochondrial mechanisms.

## 4. Addressing Model Limitations

### 4.1. PD and Environmental Factors: Neurotoxin Models and Gene Interactions

PD is a multifactorial disorder caused by complex interactions among genetic, environmental, and lifestyle factors. The influence of environmental factors on PD is significant but often overlooked. Exposure to certain environmental factors, such as pesticides, has been strongly linked to increased PD risk [7,268,269]. Based on the summary of previous research progress, we found that certain single-gene mouse models of PD, including *PRKN*, *PINK1*, and *DJ-1*, do not fully and stably recapitulate the core phenotypic features of the disease. Combining different models to study the interaction between environmental factors and PD-associated genes, either individually or in combination, provides new insights into the disease. In peer-reviewed publications, neurotoxin-induced PD mouse models occupy the largest proportion of published animal research articles. These models include those induced by 6-hydroxydopamine (6-OHDA), 1-methyl-4-phenyl-1,2,3,6-tetrahydropyridine (MPTP), pesticides, and other neurotoxins [28,270].

MPTP, 6-OHDA, rotenone, and paraquat are the most commonly used neurotoxins in PD research to induce PD-like pathological phenotypes in mouse models. MPTP crosses the blood–brain barrier rapidly due to its high lipophilicity [271,272]. Its active metabolite 1-methyl-4-phenylpyridinium has a high affinity for dopamine, norepinephrine, and serotonin transporters on the plasma membrane, allowing it to accumulate in dopaminergic neurons. Once inside the cell, MPP^+^ accumulates in the mitochondrial matrix, inducing adenosine triphosphate (ATP) depletion and oxidative stress, which cause mitochondrial dysfunction and eventually neuronal death [273,274,275,276]. Similarly, 6-OHDA, a dopamine analog, is taken up by dopaminergic and noradrenergic neurons, where it causes neuronal damage through oxidative stress and iron-mediated catalysis [277]. Rotenone is a highly toxic chemical. It functions as a potent mitochondrial complex I inhibitor, inducing oxidative stress and cell death by inhibiting ATP synthesis [270,278]. Paraquat is also a highly toxic herbicide. It selectively induces neuronal cell death in the SNpc by participating in redox cycling and activating mitochondrial apoptosis [270]. These neurotoxins provide valuable tools for modeling PD pathology and studying its underlying mechanisms in rodents.

#### 4.1.1. Interactions Between MPTP and *SNCA*

Several studies have examined how *SNCA* mutations influence susceptibility to MPTP. Nieto and colleagues reported that a C57BL/6J × SJL background heterozygous transgene-positive mice expressing *SNCA*^p.A30P^ exhibited significantly higher mortality compared to WT mice following MPTP exposure, with all deaths occurring in males—consistent with gender differences observed in PD prevalence [279]. However, subsequent studies showed that compared with the corresponding WT controls, transgenic mouse lines expressing *SNCA*^p.A53T^ or *SNCA*^p.A30P^ mutations, including C57BL/6-Tg(Thy-1-SNCA*A30P), C57BL/6-Tg(Prnp-SNCA*A30P), C57BL/6-Tg(Prnp-SNCA*A53T) and AAV- *SNCA*^p.A53T^ in C57BL/6 mice, did not exhibit a significant increase in dopaminergic neuron loss in SNpc and striatum due to MPTP exposure [280,281,282]. Despite the lack of significant dopaminergic neurodegeneration, apoptotic markers were elevated. For example, expression of B-cell lymphoma 2 (Bcl-2) and Bcl-2–Associated X Protein (Bax) in the nucleus basalis magnocellularis-substantia innominate was significantly higher in C57BL/6-Tg(Thy-1-SNCA*A30P) mice expressing *SNCA*^p.A30P^ than in WT mice following MPTP treatment [283,284]. In addition to affecting neuron survival, *SNCA* mutations also impair neuronal regeneration and dopamine metabolism. Transgenic *SNCA* expression significantly impaired the regeneration of dopaminergic neurons and fibers after MPTP treatment in both C57BL/6-Tg(Thy-1-SNCA*A30P) mice and *SNCA*^p.A53T^ transgenic mice on a mixed Swiss Webster × C57BL/6/DBA background [285,286]. Notably, high-performance liquid chromatography-electrochemical analysis revealed a more pronounced decrease in dopamine levels in the olfactory bulbs of a mixed background (Swiss Webster × C57BL/6/DBA) *SNCA*^p.A53T^ transgenic mice compared to WT mice after MPTP treatment [287].

#### 4.1.2. Interactions Between MPTP and *LRRK2*/*PINK1*

The interaction between MPTP and LRRK2 is particularly noteworthy. Similar to *SNCA*, expression of both WT *LRRK2* and *LRRK2*^p.G2019S^ on a hybrid C3H/B6 background leads to increased mortality when exposed to a standard dose of 10 mg/kg MPTP [288]. Neurodegeneration severity directly correlates with the level of *LRRK2* expression, irrespective of the specific variants [191,288,289], supporting a gain-of-function role for *LRRK2* variants in PD. Functionally, *LRRK2*^p.G2019S^ mice (MoPrP promoter-driven, B6/C3H background) showed reduced motor performance on the rotarod after MPTP treatment compared to WT mice, indicating a synergistic gene-environment effect [288]. Similarly, *PINK1* loss-of-function variants increase neuronal vulnerability to MPTP. In an in vivo study using recombinant adenoviral delivery in C57BL/6 mice, four groups were generated to express WT *Pink1*, kinase-dead (K219M), PD-associated (G309D) mutants, or a GFP control in the SN. Following MPTP treatment, only WT *Pink1* expression conferred significant neuroprotection compared with the control group, whereas both the K219M and G309D mutants failed to provide protection [290]. This highlights the protective role of PINK1 in mitigating MPTP-induced neuronal damage.

#### 4.1.3. Interactions Between Other Neurotoxins and Genetic Mutations

Similar to the results reported on the combined effects of *SNCA* mutation and MPTP exposure, on a C57BL/6J × SJL background, heterozygous mice expressing *SNCA*^p.A30P^ exhibited an increased mortality rate compared to WT controls when exposed to rotenone, despite showing no significant differences in dopaminergic neuron death [279]. However, exposure to other neurotoxins, such as 6-OHDA and paraquat, showed variability in their interactions with *SNCA* gene variants. For example, AAV-mediated expression of *SNCA*^p.A30P^ in C57BL/6 mice resulted in more pronounced 6-OHDA–induced reductions in TH fiber density in the midbrain compared with GFP vector control [291]. When exposed to paraquat and maneb, transgenic mice expressing WT *SNCA* on a C57BL/6 background significantly exacerbated loss of neural progenitors [292].

The mutations not only affect the neurons’ susceptibility to neurotoxins but also influence which brain regions are impacted. For example, under the treatments of rotenone, transgenic mice of C57BL6 background expressing *SNCA*^p.A30P^ older than 9 months exhibited a more significant reduction in hippocampal-cortical network gamma oscillations, which is an electrophysiological marker of attention and memory, compared to WT mice [293]. Additionally, in C57BL/6;C3H-Tg(MoPrP-SNCA*A53T)M83 mice, the interaction of A53T α-synuclein and paraquat treatment led to significantly enhanced α-synuclein pathologies in the cerebellar cortex, hippocampus, somatosensory and auditory cortices [294].

Other PD-linked genes also modulate neurotoxins sensitivity. For instance, in C57BL/6 J *Lrrk2*^p.G2019S^ knock-in mice, the mutant protein can further exacerbate rotenone-induced mitochondrial dysfunction and impaired neurotransmission [295]. Moreover, studies using SN neurons dissected from *Pink1* knockout mice on a C57BL/6J × 129/Sv background highlighted the essential role of PINK1 in maintaining normal mitochondrial membrane potential and protecting against neurodegeneration under rotenone treatment [296].

### 4.2. The Potential Interactions of Multiple PD Genes

The evidence summarized above demonstrates that when exposed to exogenous neurotoxins, mice with PD-related gene mutations exhibit worse outcomes than WT mice. These outcomes include higher mortality, increased neurodegeneration, impaired neurogenesis and neurotransmission, and deficits in the motor system. A critical next step is to examine potential interactions between different PARK genes. Accumulation of insoluble misfolded α-synuclein proteins can cause the attenuation of proteasome activity, lysosomal dysfunction, blocking of tubulin polymerization, inhibition of mitochondrial complex I and oxidative stress [19,114]. Therefore, it is hypothesized that *SNCA* causal variants that induce aggregation of α-synuclein may have a high likelihood of interacting with the pathological effects of other PARK gene mutations.

Initially, aggregated α-synuclein may trigger proteasome- and lysosome-mediated cellular protein degradation pathways. As previously noted, LRRK2 is also involved in regulating the function of lysosomal proteases in *Lrrk2*^p.G2019S^ knock-in C57BL/6N Tac mice [211]. When these degradation systems are overwhelmed by excess misfolded proteins, cellular health is further compromised. The inhibition of mitochondrial complex I by α-synuclein and the resulting oxidative stress may interact with proteins Parkin, PINK1, and DJ-1 [116,216,217,219,241,242,267]. Increased expression of α-synuclein as been linked to elevated levels of PINK1 protein, contributing to enhanced pS65-Ub-mediated mitophagy [297]. DJ-1 may also have connections with α-synuclein via its role in apoptosis, astrocyte proliferation, and inflammation [265,266,298,299].

Beside the synergistic effect with *SNCA*, there is emerging evidence for potential interactions among other PARK genes. PINK1 and LRRK2 both contribute to the morphological development of primary cilia in striatal neurons and cholinergic neurons [192,193,250]. Additionally, glutamatergic synaptic transmission requires the involvement of both LRRK2 and Parkin [213,227]. These results indicate the pathway connections and functional convergence among PD-related genes themselves and suggest that multiple interconnected molecular pathways underlie PD pathogenesis. In line with these observations, we have systematically summarized the relevant genetic models in Table 5 which provides a comprehensive overview of their genetic backgrounds and experimental parameters. Therefore, the application of combined transgenic models, such as double knockout and multiple mutations, is becoming a popular approach moving forward.

## 5. Evaluating Behavioral Alignment

When evaluating a novel animal model of PD, a crucial criterion is assessing the model’s ability to faithfully reproduce the hallmarks of the disease, including both pathological and neurological features. Compared with pathological analysis, behavioral testing offers the advantages of being repeatable, non-invasive, and capable of dynamic monitoring. The presence of behavioral abnormalities serves as an important indicator, typically reflecting that the relevant neurons have sustained pathological damage, potentially surpassing a critical threshold. The cardinal clinical motor manifestations of PD include bradykinesia, resting tremor, rigidity, and postural instability [3,312]. Consequently, current behavioral assessments in PD models heavily emphasize motor dysfunction, reflecting their ease of quantification and alignment with diagnostic criteria for parkinsonism. However, this emphasis risks overlooking early prodromal symptoms and non-motor comorbidities, including sleep disorder, olfactory dysfunction, autonomic dysfunction, cognition impairment and psychiatric symptoms, that define PD’s preclinical and clinical trajectory in humans. Table 6 summarizes the mouse behavioral tests which are widely used to evaluate both motor and non-motor symptoms associated with PD. The following sections described the specific manifestations of frequently reported symptoms in PD patients and the corresponding behavioral assessments in mouse models. The tested capacities and parameters were summarized to illustrate how behavioral tests are used to quantify outcomes of animal behavior. By bridging gaps between clinical PD manifestations and preclinical modeling, this integrated analysis aims to recommend appropriate behavioral phenotyping strategies, ultimately strengthening the translational power of PD mouse models.

### 5.1. Motor Symptoms and Behavioral Assessments

#### 5.1.1. Assessing Bradykinesia and Tremor in PD Models

A variety of assessments have been developed to measure motor deficits based on performance evaluation. Rest tremor, characterized by involuntary shaking of a limb or head at rest, is a cardinal motor symptom of PD. Visual observation in daily checks can be used to assess tremors without the need for a specialized behavioral assessment. Bradykinesia, which refers to slowness of movement and difficulty initiating movements, is another cardinal motor symptom of PD. Locomotor function is often used as an indicator of bradykinesia. The open field test, where mice are placed in an open arena to freely explore, is a commonly used assessment to determine levels of locomotor activity, general anxiety levels, and exploratory tendencies [313,314]. Various open field test arenas, such as home-cage, square-arena, and round-arena open fields, can be used as long as the mice can freely move and explore around [342,343,344]. Nevertheless, compared with the home-cage open field test, the standard arenas can provide more locomotor space without limitations and support the collection of more behavioral readouts [345]. Parameters such as total travel distance and average speed are notable indicators of bradykinesia. Additionally, the open field test can provide insights into the anxiety levels of mice based on their thigmotaxis (tendency to stay close to walls) behavior [346,347].

#### 5.1.2. Evaluating Muscle Strength, Rigidity and Comprehensive Motor Capacity

Rigidity is also one of the cardinal motor symptoms of PD. The characteristic rigidity is characterized by intensified muscle stiffness and evident resistance to passive movement. It manifests as a persistent hindrance to limb mobility, resulting in rigid and inflexible muscle tone. Patients with PD commonly exhibit mechanical muscle dysfunction as well, primarily reflected by reductions in muscle strength, power, and rate of force development [348]. These symptoms affect multiple muscle groups, making the initiation and execution of voluntary movements challenging. In the wire mesh grip strength test, or hanging test, mice are simply required to hang onto an upside-down mesh for as long as possible, and the hanging time is used to assess muscle strength and motor deficits [81]. PD neurotoxin-induced mice show significant grip strength deficits in this test [318]. To assess rigidity and overall motor function, tests like the pole test and rotarod are commonly employed. The pole test involves placing a mouse face-up on a rough-surfaced pole, and the time it takes for the mouse to reorient itself and land on the ground reflects its overall motor capacity [320,321]. Compared with control mice, PD mice with motor deficits needed more than 50% more time to complete the reorientation and landing [318,319,322]. The accelerating rotarod test assesses motor coordination, learning, and cardiopulmonary endurance. Mice are placed on a rotating rod that gradually increases in speed. Time spent on the rotarod reflects their balance, coordination, and endurance [316,317]. PD mice generally show reduced latency to fall due to motor impairment [318,319].

#### 5.1.3. Effective Assessment of Balance

Postural instability (along with freezing of gait) is the last cardinal motor symptoms of PD. It refers to impairments in controlling postural reflexes and body balance, which significantly increases the risk of falls [312]. Beam walking test is a effective assessment typically employed for evaluating balance and strength. In the beam walking test, mice walk across a narrow beam to reach a darkened safety box, thereby their dynamic balance and motor coordination are evaluated. Performance is quantified by the time it takes for the mouse to reach the dark box and the number of foot slips during the assessment [317,323,324]. Increased beam crossing latency and the number of foot slips indicate significant motor deficits in PD mouse models [318,322].

Quantifying the multidimensional features of gait can significantly improve the sensitivity of gait analysis, provide more precise indicators for disease progression monitoring, and offer valuable insights into the underlying mechanisms of postural instability [349]. There has been significant progress in the development of technologies capable of comprehensively evaluating freezing of gait. Two notable digital platforms in this regard are DigiGait [327] and CatWalk [350], which enable an objective assessment of motor functions by yielding an array of gait-related parameters. Both tests analyze posture and kinematics of mice through dynamic fingerprint signals generated from all four limbs, thereby offering valuable information about asymmetry and gait variability related to strength, balance, and coordination [325,326,351].

### 5.2. Non-Motor Symptoms and Behavioral Assessments

#### 5.2.1. Measuring Sleep Disorder

Certain assessments have been developed to measure non-motor deficits based on performance evaluation. It is evident that modulating sleep and circadian rhythms can effectively address disease progression in PD [352]. Sleep disorders in PD patients include excessive daytime sleepiness, insomnia, and fragmented sleep during the night [353]. Electrophysiological studies have indicated that dopaminergic neurons mediate arousal behavior through communication with the striatum, basal forebrain, and cerebral cortex [354]. In mouse models, the implantation of electroencephalogram and electromyogram electrodes is commonly used to record and quantify the sleep–wake cycle [328,329].

#### 5.2.2. Assessing Olfactory Dysfunction

Approximately 90% of PD patients experience olfactory impairments, including difficulties in identifying, detecting and discriminating odors [355]. Olfactory decline has been suggested as an indicator of neurodegeneration beyond the striatum in PD [356]. The buried food-seeking test is an assessment designed to evaluate mouse olfaction, where the time taken by the mouse to find buried food under bedding can be used as a measure of olfactory capacity [330,331,332]. Furthermore, fasting before the buried food-seeking test has been shown to enhance the performance of mice in the assessment, improving the sensitivity of the test [357].

#### 5.2.3. Evaluating Autonomic Dysfunction

PD patients are often troubled by prodromal symptoms, such as constipation, urinary incontinence, and gastrointestinal dysfunction, sometimes many years before formal clinical diagnosis [18,25,358]. Various methods are available to monitor urinary dysfunction in mouse models. For example, the voiding spot assay allows for visualizing and quantifying urinary function in mice through urine stains on filter paper, without invasive procedures [333]. The metabolic cage assay is a specialized housing unit that enables continuously monitoring and measuring various physiological parameters, including metabolic rate, food and water intake, and waste output [334].

#### 5.2.4. Measuring Psychiatric and Cognitive Symptoms

Meta-analytic studies have reported that approximately 38% of PD patients experience depression, while 40% and 26.3% are diagnosed with mild cognitive impairment and dementia, respectively [359,360,361]. Accordingly, several assessments have been designed to measure the emotional and cognitive capacities of mice. Forced swim tests continue to be widely used to assess the level of depression in mice [314,335]. The elevated maze test focuses on anxiety-related behavior and introduces fear of heights to modify the exploratory behavior of mice [336]. Various mazes, such as the T-maze, Y-maze, Barnes maze, and Morris water maze, are designed to evaluate the cognitive capacity, memory, and learning in mice. In these tests, mice are trained to remember targets under the motivation of food rewards or shelter, with the ability to recognize targets and complete memory tasks serving as key indicators of spontaneous alternation, spatial learning, memory retrieval, and cognitive flexibility [314,337,338,339,340,341].

While there continues to be much progress in the development of behavioral phenotyping methods for use to explore parkinsonian symptoms in mice, there remains a need to formalize and harmonize these methods so that direct comparisons can be made between studies.

## 6. An Optimization-Oriented Framework

### 6.1. The Impact of Experimental Variability on Data Interpretation and Reproducibility

The use of genetic mouse models has significantly advanced our understanding of the molecular mechanisms underlying PD. However, we found that even mouse models carrying the same single-gene mutation still fail to consistently recapitulate the same pathological and neurological features of PD. Based on our extensive review of literature, various experimental variables have been identified as critical contributors to significant discrepancies in the measured experimental outcomes (Table 7). These discrepancies can cloud data interpretation and limit the research community’s ability to find consensus.

For example, while C57BL/6 and FVB are the two most widely used mouse strains for genetically modified mice, the differences in genetic background and stress responses between these strains profoundly influence phenotypic outcomes, such as neurodegeneration patterns and behavioral deficits. For specific genes, unique pathogenic mutations lead to distinct protein dysfunction and further affect the specificity and severity of PD-like phenotypes. The choice of transgenic methods (e.g., PAC, BAC, AAV, CRISPR-Cas9), and promoters (e.g., PDGFB, Thy1, CMV) directly impacts transgene expression levels and spatial distribution. Moreover, although most PD-like symptoms, including motor deficits, α-synuclein aggregation, and neurodegeneration, manifest progressively in aged mice, studies are not regularly performed at different ages, emphasizing the need for longitudinal studies to better model human disease progression. Finally, the existing motor and non-motor behavioral tests lack standardized protocols, with variations in equipment design, testing parameters, and scoring criteria, complicating cross-study comparisons.

**Figure 1:**
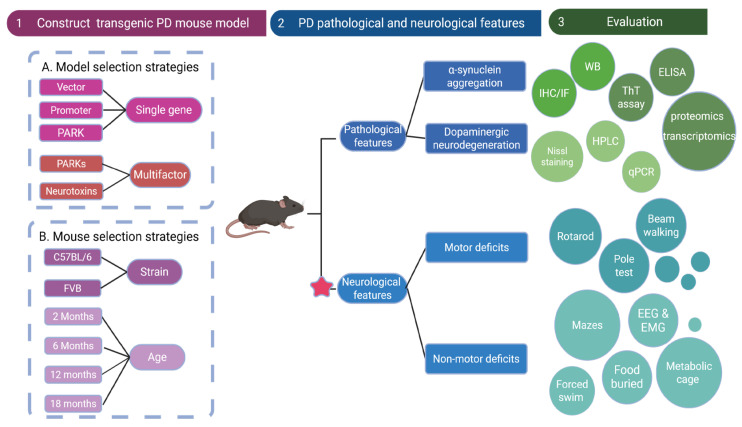
Roadmap for the construction and evaluation of Parkinson’s disease (PD) mouse models. The red star highlights the focus of this study on the optimization and standardization of experimental designs for assessing neurological features.

Taking the aforementioned variables into consideration, we have designed a comprehensive strategic roadmap (Figure 1) to provide guidelines for investigating the pathogenic mechanisms of PD using genetic mouse models, aiming to minimize variability and enhance comparability across different studies.

### 6.2. Recommended Strategies for Improving Models in the Future

Collectively, our study highlights the urgent need for a more optimized and standardized approach to the evaluation of genetic mouse models of PD. Greater harmonization of experimental practices is needed, including more careful consideration of strain selection, standardized genetic strategies, age windows, and behavioral protocols. Such improvements will enhance reproducibility, strengthen model validity, and accelerate the translation of preclinical findings to clinical applications.

One important consideration is the choice of mouse background strain and backcrossing strategy. Establishing and characterizing transgenic modifications across diverse mouse strains may provide important insights into the differential susceptibility of specific pathways to perturbation, including responses to toxic insults. A well-known example is the preference of using C57BL/6 mice in MPTP-induced PD models, largely because this strain has higher toxin susceptibility to MPTP neurotoxicity compared to other strains [362,363,364,365,366,367]. By contrast, although the FVB/N mice have been used in some *LRRK2* transgenic models, this strain has several limitations for PD modeling. These include lower sensitivity to MPTP, higher spontaneous locomotor activity, and visual defects, that may introduce considerable variability in behavioral phenotyping [188,368,369]. Genetic background is a major determinant of phenotype. C57BL/6 mice, including C57BL/6J, C57BL/6N and C57BL/6N Tac substrains, represent the most widely used genetic background in PD mouse models. Notably, significant genetic differences exist between these substrains. Single nucleotide polymorphism analyses have identified multiple genetic variations between C57BL/6J and C57BL/6N, and importantly, a loss-of-function mutation in the nicotinamide nucleotide transhydrogenase gene is present exclusively in the C57BL/6J substrain [370]. C57BL/6J and C57BL/6N mice exhibit not only genetic divergence but also pronounced phenotypic differences. Compared with C57BL/6N mice, C57BL/6J mice display higher locomotor activity, better motor coordination, and enhanced olfactory associative learning [371,372]. In addition, C57BL/6J mice show impaired insulin secretion under high-fat diet conditions and exhibit relatively higher blood pressure (approximately 10 mmHg difference) [373,374]. Therefore, a detailed description of mouse background strain and the specific genetic modifications is essential for ensuring experimental reproducibility and comparability.

However, mouse strain background and backcrossing strategies have not received sufficient attention in PD studies employing genetic mouse models. In many cases, descriptions of mouse strain background are limited, and key information regarding backcrossing procedures and the number of backcross generation is often omitted in the literature. Strain background is frequently reported in a simplified manner, using broad or ambiguous terms such as B6/129, C57BL/6, 129/Sv, or C3, without further clarification of substrain composition or breeding history. As illustrated by several studies discussed above, different research groups have employed varying backcrossing strategies, ranging from N3 to N15 generations [112,258,282,308]. This variability, combined with insufficient reporting, may lead to inconsistencies in genetic background and compromise the reproducibility and comparability of experimental findings. Therefore, standardized nomenclature and comprehensive documentation of genetically modified mouse models is essential, including, but not limited to, species, strain, substrain, stock, breeding history, genotyping records, and phenotypic characterization [375,376,377].

Common techniques for generating genetically modified mice include pronuclear microinjection, embryonic stem (ES) cell–mediated targeting, and CRISPR/Cas9 genome editing [376]. Following the generation of genetically modified mouse models, genotyping is required to confirm the presence and integrity of genetic modifications and to ensure the accuracy of the genotype of experimental animals. Polymerase chain reaction (PCR) remains the most commonly used technique, enabling efficient discrimination between heterozygous and homozygous genotypes. However, conventional PCR can only determine the presence or absence of specific alleles and does not provide information on sequence accuracy. Therefore, Sanger sequencing is typically performed to validate PCR products, allowing confirmation of the precise mutation or insertion sequence, verification of junction integrity, and exclusion of potential off-target sites and unintended mutations. In addition, mRNA expression levels can be assessed using quantitative real-time PCR (qRT-PCR). For the determination of gene copy number at the genomic DNA level, genomic quantitative PCR (qPCR) or Southern blot analysis should be employed.

As noted above, the genetic background of mice represents a critical variable influencing experimental outcomes. To minimize background-related variability, backcrossing strategies are the most widely employed technology to standardize the genetic background of genetically engineered mouse models. In conventional backcrossing, mice carrying a genetic modification are repeatedly crossed with a defined inbred recipient strain, for instance, C57BL/6J. In each generation, heterozygous carriers with the genetic background most closely matching the recipient strain are selected for subsequent backcrossing. After ten or more generations, the resulting line is considered congenic, with >99.9% of its genome derived from the recipient strain [87], thereby minimizing strain-dependent phenotypic variation.

However, conventional backcrossing is a stochastic and time-consuming process. To accelerate this process, marker-assisted backcrossing (also known as speed congenics) can be employed [88]. This approach utilizes genome-wide molecular markers, such as single nucleotide polymorphisms (SNP), to identify and select individuals with the highest proportion of the desired genetic background at each generation, achieving >99% background purity within approximately 5–6 generations [89]. Speed congenics can save approximately four weeks per generation and requires fewer generations overall, thereby substantially reducing total time, cost, and animal usage [89]. Although speed congenics substantially reduces breeding time, it is essential to explicitly report its use, as residual donor genomic regions may persist and potentially influence phenotypic interpretation.

After the establishment of genetically engineered mouse lines, genetic monitoring is an essential component of quality assurance in laboratory animal research, ensuring genetic integrity, stability, and reproducibility across studies. Over time, genetic drift, spontaneous mutations, and unintended crossbreeding with other strains may occur during colony maintenance, leading to genetic contamination and potentially affecting experimental outcomes [376,377]. To mitigate these risks, routine genetic monitoring of breeding animals should be performed using defined genetic markers (e.g., SNP molecular markers) or sequencing-based approaches [376,377]. It is also recommended that colonies be periodically refreshed from cryopreserved stocks to preserve genetic integrity [377]. Furthermore, strict colony management practices, such as physical separation of different strains and the use of standardized breeding protocols, are essential to minimize contamination and ensure experimental consistency.

Consistent age stratification is another important priority. Mouse life stages can be broadly divided into five stages: juvenile, young adult, mature adult, middle-aged, and aged. In young adult period, sexual maturity is typically attained at around 35 days of age, whereas full systemic immune maturation occurs by the eighth week [378,379]. The mature adult period is generally considered to range from 3 to 6 months of age, based on the rate of maturational growth and the appearance of age-related biomarkers [379,380]. The middle-aged period and aged period are generally estimated to occur at approximately range from 10 to 14 months and 18 to 24 months of age, respectively, based on senescence-associated changes, such as collagen cross-linking and accumulation of activated/memory T cells [380]. To improve consistency and to better capture progressive phenotypes, behavioral tests should be conducted at specific time points corresponding to major developmental stages, such as 2, 6, 12, and 18 months of age.

In preclinical studies of PD, male mice are predominantly used, whereas female animals are often underrepresented. Consistently, among the models summarized in this review, female mice are rarely included, with most studies relying almost exclusively on male cohorts. It is primarily influenced by several factors. First, epidemiological evidence demonstrates sex-related differences in both the prevalence and incidence of PD. In general, the number of male patients is approximately 20% higher than that of female patients, and disease onset tends to occur earlier in men than in women [381,382]. Second, in toxin-induced models of PD, such as those based on MPTP, estrogen has been shown to exert neuroprotective effects [383,384]. Consequently, female mice often display reduced susceptibility to dopaminergic neurodegeneration, whereas male mice tend to exhibit more robust and consistent phenotypic outcomes [383,384]. Female mice undergo estrous cycle-related hormonal fluctuations, which may increase variability in behavioral and neurochemical outcomes. In contrast, male mice tend to exhibit more stable and consistent performance in behavioral assays, such as the rotarod tests [385].

In addition, behavioral neuroscience experiments are inherently influenced by biological, environmental, and operational variables, making complete uniformity difficult to achieve. Nevertheless, rigorous adherence to standard operating procedures and comprehensive reporting of relevant variables are essential for ensuring the validity and reliability of experimental findings. As a practical strategy, we recommend adopting standardized operating procedures that have been validated in previous studies [314,323,325,331,340,386,387]. Some variables are not mentioned in standard operating procedures, but their influence on experimental results remains equally important to consider. For example, providing sufficient habituation and training before testing can reduce novelty-induced stress and improve data reliability. Similarly, familiarizing mice with non-aversive handling methods, such as tunnel or cup handling, has been shown to significantly reduce stress and anxiety and may improve performance in behavioral tests [388,389]. Body weight and circadian rhythm are also critical factors that must be carefully monitored. Body weight can influence behavioral performance in mice, particularly in measures related to locomotion and motor coordination [390], and should therefore be monitored, balanced where possible between groups, and accounted for during data interpretation. Circadian rhythm is another critical variable, as mice are nocturnal animals, exhibiting substantially higher spontaneous activity during the dark phase [391]. Therefore, to ensure comparability between experimental groups, all behavioral tests should be conducted within a consistent time window each day to eliminate systematic biases introduced by circadian variation. Incorporating and controlling a broader range of variables can establish a foundation for cross-model comparisons and enhance the reproducibility of experimental results. More robust and transparent experimental frameworks will also facilitate collaborative refinement of methods and enable more straightforward comparisons between studies.

## 7. Conclusions

In this review, we systematically summarize recent advances in transgenic mouse models of the PARK gene family. Our analysis indicates that *SNCA*- and *LRRK2*-related models recapitulate, to some extent, the core pathological features and neurological symptoms of PD, making them suitable for subsequent therapeutic studies. In contrast, *PRKN*, *PINK1*, and *DJ1* transgenic mice only partially recapitulate dopaminergic neurodegeneration and motor deficits; these phenotypes are often inconsistent and lack stability across studies. Moreover, these models generally fail to reproduce α-synuclein aggregation and show limited ability to capture non-motor behavioral phenotypes. Future strategies incorporating gene-neurotoxin combination or multigenic approaches may help better elucidate the specific roles that individual PARK genes play in the onset and progression of PD, as well as the interactive pathogenic mechanisms that might be involved. Such efforts would deepen our understanding of disease pathogenesis.

In addition, based on the core clinical features of PD, we review commonly used behavioral testing paradigms that could be used to evaluate the utility of these models. Building on the above analysis, we further propose a systematic experimental design roadmap aimed at improving control of key variables and providing methodological guidance for studies leveraging transgenic models to dissect PD mechanisms. Ultimately, a rigorous and standardized approach for the development and evaluation of transgenic PD models will enhance the translational value of preclinical studies and contribute to the development of interventions that benefit patients with PD.

## Figures and Tables

**Table 1:** Summary of common pathogenic mutations in the five most studied familial PD genes.

Gene	Protein and Related Pathway	Mutation	Mutation Effects
*SNCA*	*SNCA*-encoded α-synuclein is a presynaptic neuronal protein involved in synaptic vesicle trafficking and neurotransmitter release. In PD, misfolded α-synuclein aggregates to form Lewy bodies, a pathological hallmark of PD.	A30P	Promote the insoluble aggregation of α-synuclein [36]
		E46K	Promote the insoluble aggregation of α-synuclein [37,38]
		H50Q	Promote the insoluble aggregation of α-synuclein [39]
		G51D	Increase the toxicity of aggregated fibrils [40]
		A53T/E	Promote the insoluble aggregation of α-synuclein [36,41]
		Duplication/Triplication	Promote the increased expression of α-synuclein [42,43]
*LRRK2*	*LRRK2*-encoded leucine rich repeat kinase 2 (LRRK2) is a multifunctional kinase involved in intracellular signaling, vesicle trafficking, autophagy, and cytoskeletal dynamics. Dysfunctional LRRK2 leads to neuronal toxicity through abnormal phosphorylation of downstream targets, causing impaired autophagy, mitochondrial dysfunction, and increased α-synuclein accumulation.	N1437H	Decrease GTPase activity [44]
		R1441C/G/H	Decrease GTPase activity [45,46]
		Y1699C	Decrease GTPase activity [47]
		G2019S	Increase central kinase domain activity [48]
		I2020T	Increase central kinase domain and GTPase domain activity [49]
*PRKN*	*PRKN*-encoded Parkin acts as an E3 ubiquitin-protein ligase that tags damaged proteins and mitochondria for degradation via the ubiquitin-proteasome system and mitophagy. Dysfunctional Parkin results in mitochondrial dysfunction, impaired mitophagy, oxidative stress.	R42P	Decrease protein structure stability [50,51]
		K48A	Decrease protein–protein interactions [50,51]
		T240R	Nonsense mutation in exon 6, loss of function [52,53]
		R275W	Decrease protein structure stability [54]
		Q311Ter	Nonsense mutation in exon 8, loss of function [52,53]
*PINK1*	*PINK1*-encoded PTEN induced kinase 1 (PINK1) is a mitochondrial kinase that detects mitochondrial damage and recruits Parkin to initiate mitophagy. Dysfunctional PINK1 leads to defective mitophagy, mitochondrial depolarization, increased oxidative damage, resulting in accumulation of damaged mitochondria and neuronal stress in PD.	G309D	Decrease kinase activity and dysregulate mitophagy [55,56,57,58]
		T313M	Inhibit phosphorylation [59]
		L347P	Decrease protein stability [60] and increase degradation [61]
		G411S	Decrease kinase activity [62]
		W437Ter	Lack the C-terminus and part of the kinase domain, loss of mitophagy regultion [57]
		Q456Ter	Decrease protein expression level and kinase activity [62]
*DJ-1*	*DJ-1*-encoded protein parkinsonism associated deglycase (DJ-1) functions as an oxidative stress sensor and antioxidant, protecting cells from oxidative stress and regulating mitochondrial function. Dysfunctional DJ-1 impairs antioxidant defense, increasing oxidative damage, particularly in dopaminergic neurons.	L10P	Decrease protein stability [63]
		M26I	Decreases protein expression levels [64,65]
		Q45Ter	Nonsense mutation in exon 8, loss of function [66]
		A104T	Decreases protein stability [67] and increases degradation [64]
		P158del	Decrease protein stability [63]
		L166P	Decrease protein stability, increase protein degradation [64,67,68,69]

**Table 2:** Summary of transgenic mouse models of PD associated with PARK genes with less frequent PD-associated mutations.

Gene	Genetic Model	Mutation Effects	PD-Like Pathologies and Phenotypes
PARK*5*	*UCH-L1* ^I93M^	Reduction in hydrolase activity [70]	Dopaminergic neurodegeneration [71] Promote tubulin polymerization [72]
	*NT-UCH-L1*	N terminal cutting, induce aggregation	Tendency to be monoubiquitinated and readily aggregated [73]
PARK*9*	*Atp13a2* ^−/−^ *(AAV-Cre)* *Atp13a2* ^−/−^	Knockout, loss of function	Dopaminergic neurodegeneration [74] No PD-like neuropathology [75,76] No motor deficit but decreased spontaneous movement [75] Gliosis in brain, lipofuscinosis, and endolysosomal abnormalities [75]
PARK*11*	*Gigyf2* ^−/−^ *Gigyf2* ^+/−^	Knockout, loss of function	Die within the first 2 post-natal days [77] Motor dysfunction [77]
PARK*13*	MND2 *(Htra2*^−/−^*)*	Knockout, loss of function	Brief lifespan (40 days) with organ hypoplasia and muscle wasting [78,79,80,81] Neurodegeneration and oligomeric α-synuclein aggregation [79,80,82] Abnormal neural electrical activity and neuroinflammation [83,84]
PARK*14*	*Pla2g6* ^−/−^	Knockout, loss of function	Shorter lifespan [85] Dopaminergic and axonal neurodegeneration and striatal α-synuclein accumulation [86,87,88] Motor deficits [86,87]
	*Pla2g6* ^D331Y^	Decrease phospholipase activity [89]	Dopaminergic neurodegeneration [90] Mitochondrial dysfunction, endoplasmic reticulum (ER) stress, and mitophagy impairment [90]
	*Pla2g6* ^G373R^	No glycerophospholipid catalyzing enzyme [91]	Dopaminergic neurodegeneration [88] Motor deficit [88]
PARK*17*	*V* *ps* *35* ^−/−^	Knockout, loss of function	Early embryonic lethality [92] Rod cell death [93]
	*V* *ps* *35* ^+/−^	N/A	Corneal dystrophy [94]
	*VPS35* ^D620N^	Inhibit autophagy [95]	Dopaminergic neurodegeneration and α-synuclein aggregation [96] Motor deficit [96] Mitochondrial dysfunction and hippocampal neurogenesis impairment [96,97]
PARK*19*	*D* *najc* *6* ^−/−^	Knockout, loss of function	Impaired pre-synaptic plasticity in the primary visual cortex [98]
PARK*20*	*S* *ynj* *1* ^−/−^	Knockout, loss of function	Brief lifespan [99]
	*S* *ynj1* ^+/−^	Impaired 5′-phosphatase activity	Reduction in dopaminergic terminals [99] Hyperactivity and motor deficit [99]
	*S* *ynj1* ^R258Q^	Impaired Sac1 domain phosphatase activity [100]	60% survival rate [100] No dopaminergic neurodegeneration but morphological abnormality [100]

**Table 3:** Recent additions to the collection of *SNCA*, *LRRK2*, *PRKN*, *PINK1* and *DJ-1* genetic rodent PD and their assessed phenotypes.

Phenotype	Gene				
Assessed	*SNCA*	*LRRK2*	*PRKN*	*PINK1*	*DJ1*
Neurodegeneration	OE-WT, A53T	R1441G, R1441C [102], G2019S	Q311Ter	-----	Exon 2 KO, Exon 3–5 KO
Synucleinopathy	OE-WT, A30P, A53T, E46K	G2019S, R1441G [103]	Q311Ter	G309D	-----
Motor deficits	OE-WT, hA30P, A53T, E46K	R1441G, R1441C [102], G2019S	S65A [104], R275W [105], Q311Ter	G309D	Exon 2 KO, Exon 3–5 KO
Sleep disorder	OE-WT, A53T [106]	G2019S	-----	-----	-----
Olfactory dysfunction	OE-WT, A30P, A53T [107]	KO [108]	-----	-----	-----
Autonomic dysfunction	OE-WT, A30P, A53T	R1441G/C, R1628P [109], G2019S [110]	-----	-----	-----
Psychiatric and cognitive symptoms	OE-WT, A53T	G2019S [111]	Exon 3 KO	Exon 4–5 KO [112]	-----

Abbreviations: OE-WT: Overexpression of WT gene; KO: knockout.

**Table 4:** Examples of PD treatment developments targeting α-synuclein.

Treatments	Involved Mechanism	Pathway/Function
1. Exosome-mediated antisense oligonucleotide 4	Expression	1. Blocks the expression of *SNCA* specifically in vivo and vitro [148]
2. Indatraline-conjugated antisense oligonucleotide		2. Attenuates the production of α-synuclein and related dopamine dysfunction [149,150]
3. Posiphen		3. Recognizes *SNCA* messenger RNA (mRNA) and inhibits the expression of α-synuclein [136]
4. Nano-MgO micelle composite- α-synuclein-mRNA		4. Crosses the BBB to target the neurons and attenuates the expression of α-synuclein [151]
1. Syn9048 (pan-α-synuclein antibody)	Transmission	1. Decreases the α-synuclein pathology in several brain regions by selectively binding to pathogenic α-synuclein, inhibiting its cell-to-cell transmission and promoting its clearance [152]
2. BIIB054 (human-derived α-syn antibody)		2. Binds with α-synuclein via high affinity to rescue the dopamine transporter loss and motor deficits [153]
3. Toll-like receptor 2		3. Blocks the transmission of α-synuclein between neuron-neuron and neuron-astrocyte [154]
1. Eicosanoyl-5-hydroxytryptamide	Aggregation	1. Activates the dephosphorylation of α-synuclein to reduce the protein fibrillation [155]
1. Felodipine	Degradation	1. Enhances autophagy to degrade α-synuclein aggregates [156]
2. Tat-βsyn-degron		2. A peptide that can cross the BBB and plasma membrane to knockdown α-synuclein [157]

**Table 5:** Summary of reporting details for genetically engineered mouse models.

GEM	GEM Name Interpreted from the Publication: Background (Breeder) and the WT Strain Used as a Control	Details		Comments	Ref.
*SNCA*	h*SNCA* × *Cebpb^tm1Vpo^*/J (JAX 006873) https://www.jax.org/strain/006873 h*SNCA* × 129/Sv;C6J-Aep^−/−^ [300] Control: B6.Cg-Tg(SNCA)OVX37Rwm *Snca^tm1Rosl^*/J (JAX 023837) https://www.jax.org/strain/023837	Age	4 M, 8 M, 14 M	suggested control: C57BL/6J or *Cebpb*^+/+^ WT littermates	[133]
		Sex	Male & Female		
		Number	5–6		
		Backcrosses	N/A		
*SNCA*	C57BL/6J (JAX 000664) with AAV2/9-hSyn1-h*SNCA* injection, https://www.jax.org/strain/000664 Control: C57BL/6J with AAV2/9-hSyn1-EGFP injection	Age	8–9 W		[137]
		Sex	Male		
		Number	10		
		Backcrosses	N/A		
*SNCA*	C57BL/6J-Tg(BAC-SNCA-GFP) Control: C57BL/6J	Age	3 M, 8 M, 13 M		[138]
		Sex	Male		
		Number	3–4		
		Backcrosses	N/A		
*SNCA* ^p.A^ ^53T^	C57BL/6J- Tg(BAC-SNCA*A53T^+/−^) [301] Control: C57BL/6J	Age	5 M, 7 M, 9 M, 11 M, 13 M		[106]
		Sex	Male		
		Number	WT (5), TG (6)		
		Backcrosses	N > 10		
*SNCA* ^p.A^ ^53T^	B6;C3-Tg(Prnp-SNCA*A53T)83Vle/J (JAX 004479) https://www.jax.org/strain/004479 Control: C57BL/6J	Age	18 M	Suggested control: B6C3F1/J	[134]
		Sex	N/A		
		Number	5		
		Backcrosses	N/A		
*SNCA* ^p.A^ ^53T^	B6;C3-Tg(Prnp-SNCA*A53T)83Vle/J (JAX 004479) Control: B6C3F1/J (JAX 100010) https://www.jax.org/strain/100010	Age	8 M		[140]
		Sex	Male		
		Number	3–5		
		Backcrosses	N/A		
*SNCA* ^p.A^ ^53T^	FVB;129S6-Tg(SNCA*A53T)1Nbm *Snca^tm1Nbm^* Tg(SNCA*A53T)2Nbm/J (JAX 010799) https://www.jax.org/strain/010799 Control: C57BL/6J	Age	1 M, 3 M, 6 M, 12 M	FVB;129S6	[139]
		Sex	N/A		
		Number	8		
		Backcrosses	N/A		
*SNCA* ^p.A^ ^53T^	B6.Cg-*2310039L15Rik*^Tg(Prnp-SNCA*A53T)23Mkle^/J (JAX 006823) https://www.jax.org/strain/006823 Control: C57BL/6J	Age	3 M, 6 M		[107]
		Sex	Male & Female		
		Number	12		
		Backcrosses	N/A		
*SNCA* ^p.A^ ^53T^	AAV-mediated *SNCA*^A53T^ on C57BL/6 Control: AAV-mediated GFP on C57BL/6	Age	8 W		[281]
		Sex	Male		
		Number	WT (14), KI (28)		
		Backcrosses	N/A		
*SNCA* ^p.A^ ^53T^	129S6/SvEvTac;FVB/N;C57/BL6-Tg(PAC-SNCA*A53T) *Snca*^−/−^ [302] Control: 129S6/SvEvTac; FVB/N and 129S6/SvEvTac; FVB/N-*Snca*^−/−^	Age	4 M, 7 M, 9 M		[136]
		Sex	Male		
		Number	9–12		
		Backcrosses	N/A		
*SNCA* ^p.A^ ^53T^	Tg(h*SNCA*^p.A53T^) mice on a mixed background (Swiss Webster × C57BL/6/DBA F1) [303] Control: unclear	Age	10 M		[287]
		Sex	N/A		
		Number	saline (8), MPTP (10)		
		Backcrosses	N/A		
*SNCA* ^p.A^ ^53T^	C57BL/6;C3H-Tg(MoPrP-SNCA*A53T)M83 [304] Control: C57BL/6;C3H	Age	8 M, 12 M		[294]
		Sex	N/A		
		Number	WT (5), TG (7–9)		
		Backcrosses	N/A		
*SNCA* ^p.A^ ^53T^ *SNCA* ^p.^ ^A30P^	C57BL/6-Tg(Prnp-SNCA*A30P) *Snca*^−/−^, C57BL/6-Tg(Prnp-SNCA*A53T) *Snca*^−/−^ [120] Control: C57BL/6	Age	6 M		[282]
		Sex	Male		
		Number	5–6		
		Backcrosses	10		
*SNCA* ^p.^ ^A30P^	C57B/6J;SJL-Tg(PrP-SNCA*A30P) Control: C57B/6J;SJL	Age	3–4 M, 6–8 M		[279]
		Sex	Male/Female		
		Number	N/A		
		Backcrosses	N/A		
*SNCA* ^p.^ ^A30P^	C57BL/6-Tg(Thy-1-SNCA*A30P) [305] Control: C57BL/6	Age	6 M		[280]
		Sex	N/A		
		Number	N/A		
		Backcrosses	4		
*SNCA* ^p.^ ^A30P^	C57BL/6-Tg(Thy-1-SNCA*A30P) [305] Control: C57BL/6	Age	2–3 M		[284]
		Sex	Male		
		Number	N/A		
		Backcrosses	N/A		
*SNCA* ^p.^ ^A30P^	C57BL/6-Tg(Thy-1-SNCA*A30P) [305] Control: C57BL/6	Age	2–6 M, 9–11 M, 12–13 M, 14–16 M		[293]
		Sex	Male & Female		
		Number	2–6 M (21), 9–11 M (7), 12–13 M (7), 14–16 M (3)		
		Backcrosses	N/A		
*Snca* ^p.G51D^	C57BL/6J-*Snca*^p.G51D^ [306] Control: C57BL/6J	Age	3 M, 12 M		[135]
		Sex	Male & Female		
		Number	WT 7, KI 13		
		Backcrosses	N > 5		
WT h*SNCA* *LRRK2*^p.G2019S^	C57BL/6J-Tg(mthy1-SNCA) C57BL/6J-Tg(mthy1-LRRK2* G2019S) Control: C57BL/6J	Age	10 M		[292]
		Sex	N/A		
		Number	8		
		Backcrosses	N/A		
*LRRK2* ^p.R^ ^1441C^	FVB/N-Tg(PDGFβ*R1441C) [307] Control: FVB/N	Age	12 M, 16 M		[179]
		Sex	N/A		
		Number	10		
		Backcrosses	N/A		
*LRRK2* ^p.R^ ^1441C^	FVB/N-Tg(PDGFβ*R1441C) [307] Control: FVB/N	Age	N/A		[210]
		Sex	N/A		
		Number	N/A		
		Backcrosses	N/A		
*LRRK2* ^p.R^ ^1441C^	B6.Cg-*Lrrk2^tm1.1Shn^*/J, (JAX 009346) https://www.jax.org/strain/009346 Control: C57BL/6 J	Age	7 M		[192]
		Sex	N/A		
		Number	4		
		Backcrosses	N/A		
*LRRK2* ^p.R^ ^1441C^	B6.Cg-*Lrrk2^tm1.1Shn^*/J (JAX 009346) Control: C57BL/6 J	Age	10 W		[193]
		Sex	Male & Female		
		Number	3		
		Backcrosses	N/A		
*LRRK2* ^p.R^ ^1441G^	FVB/N-Tg(LRRK2*R1441G)135Cjli/J Control: FVB/N	Age	9–10 M, 16 M		[187]
		Sex	Male		
		Number	N/A		
		Backcrosses	N/A		
*LRRK2* ^p.R^ ^1441C^	C57BL/6J.C3H/HeJ-Tg(PDGFβ-LRRK2* R1441C) Control: C57BL/6J	Age	6 M, 15 M		[102]
		Sex	Male		
		Number	6–9		
		Backcrosses	3–4		
*LRRK2*^p.G2019S^ *LRRK2*^p.R1441C^	C57BL/6J.C3H/HeJ-Tg(PDGFβ-LRRK2*G2019S) C57BL/6J.C3H/HeJ-Tg(PDGFβ-LRRK2*R1441C) Control: C57BL/6J	Age	15–21 M		[102]
		Sex	N/A		
		Number	WT (8–9), TG (4–6)		
		Backcrosses	3–4		
*LRRK2* ^p.G^ ^2019S^	C57BL/6J-Tg(LRRK2*G2019S)2AMjff/J (JAX:018785) https://www.jax.org/strain/018785 Control: C57BL/6J	Age	2–4.5 M, 10–12 M, 15 M, 20–21 M		[195]
		Sex	Male/Female		
		Number	4–23		
		Backcrosses	Y		
*LRRK2* ^p.G^ ^2019S^	C57BL/6J-Tg(LRRK2*G2019S)2AMjff/J (JAX 018785) Control: C57BL/6J	Age	2 M, 10 M		[203]
		Sex	Male		
		Number	3		
		Backcrosses	N/A		
*LRRK2* ^p.G^ ^2019S^	C57BL/6J-Tg(LRRK2*G2019S)2AMjff/J hemizygous mice (JAX 018785) Control: C57BL/6J	Age	10–12 M		[207]
		Sex	Male & Female		
		Number	WT (8), TG (9)		
		Backcrosses	N/A		
*LRRK2* ^p.G^ ^2019S^	C57BL/6J-Tg(LRRK2*G2019S)2AMjff/J hemizygous mice (JAX 018785) Control: C57BL/6J	Age	10–12 M		[208]
		Sex	Male & Female		
		Number	WT (4), TG (7)		
		Backcrosses	N/A		
*LRRK2* ^p.G^ ^2019S^	B6;C3-Tg(PDGFβ-LRRK2*G2019S)340Djmo/J (JAX 016575) https://www.jax.org/strain/016575 Control: C57BL/6J (speculated) [102]	Age	65–83 W		[196]
		Sex	Male & Female		
		Number	WT (5), TG (10)		
		Backcrosses	N/A		
*LRRK2* ^p.G^ ^2019S^	C57BL/6-*Lrrk2^tm4.1Arte^* (Taconic 13940) Control: C57BL/6NTac	Age	N/A		[212]
		Sex	N/A		
		Number	3		
		Backcrosses	N/A		
*LRRK2* ^p.G^ ^2019S^	G2019S-*LRRK2* transgenic mice (JAX Unclear) Control: WT-*LRRK2* transgenic mice (JAX Unclear)	Age	3 M		[194]
		Sex	Male		
		Number	5		
		Backcrosses	N/A		
*LRRK2* ^p.G^ ^2019S^	C3H;B6-Tg(MoPrp-LRRK2*G2019S) Control: B6C3F1	Age	4 M, 9 M, 12 M		[288]
		Sex	Male & Female		
		Number	24		
		Backcrosses	N/A		
*LRRK2* ^p.G^ ^2019S^	C57BL/6-*Lrrk2^tm4.1Arte^*, (Taconic 13940) https://www.taconic.com/products/mouse-rat/gems/live-gems/lrrk2-g2019s-mouse C57BL/6J-Tg(BAC-LRRK2*G2019S) [182] Control: Unclear	Age	13 M	Suggested control: C57BL/6NTac	[193]
		Sex	Male & Female		
		Number	4–5		
		Backcrosses	N/A		
*LRRK2* ^p.G^ ^2019S^	FVB/N-Tg(LRRK2*G2019S)1Cjli/J (JAX 009609) https://www.jax.org/strain/009609 Control: FVB/NJ (JAX 001800) https://www.jax.org/strain/001800	Age	7–10 W		[111]
		Sex	Male/Female		
		Number	>10		
		Backcrosses	N/A		
*LRRK2* ^p.G^ ^2019S^	FVB/N-Tg(LRRK2*G2019S)1Cjli/J (JAX 009609) Control: FVB/N	Age	2 M, 4 M, 6 M, 8 M, 10 M	Suggested control: FVB/NJ	[110]
		Sex	N/A		
		Number	5–6		
		Backcrosses	N/A		
*LRRK2* ^p.G^ ^2019S^	FVB/N-Tg(LRRK2*G2019S)1Cjli/J (JAX 009609) Control: Not specified	Age	20–24 M	Suggested control: FVB/NJ	[200]
		Sex	Male		
		Number	WT (16), TG (17)		
		Backcrosses	N/A		
*LRRK2* ^p.G^ ^2019S^	FVB/N-Tg(LRRK2*G2019S)1Cjli/J (JAX 009609) FVB/N-Tg(LRRK2)1Cjli/J (JAX 009610) https://www.jax.org/strain/009610 Control: FVB/NJ	Age	11 M		[202]
		Sex	N/A		
		Number	5–10		
		Backcrosses	N/A		
*LRRK2* ^p.G^ ^2019S^	adenoviral vector-mediated injection on C57BL/6J Control: C57BL/6J	Age	7–8 W, 18.5 M		[201]
		Sex	Male		
		Number	N/A		
		Backcrosses	N/A		
*Lrrk2* ^p.R1628P^	C57BL/6J-*Lrrk2*^p.R1628P^ (Beijing Biocytogen) Control: C57BL/6J	Age	8 W		[109]
		Sex	Male&Female		
		Number	7		
		Backcrosses	N/A		
*Lrrk2* ^p.R1441G^	C57BL/6N-*Lrrk2*^p.R1441G^ [308] Control: C57BL/6N	Age	18 M		[103]
		Sex	Male		
		Number	Cortex (14), Striatum (8)		
		Backcrosses	7		
*Lrrk2* ^p.R1441G^	C57BL/6N-*Lrrk2*^p.R1441G^ [308] Control: C57BL/6N	Age	3 M, 18 M		[209]
		Sex	N/A		
		Number	5		
		Backcrosses	8		
*Lrrk2* ^p.G2019S^	B6.Cg-Tg(Lrrk2*G2019S)2Yue/J (JAX 012467) https://www.jax.org/strain/012467 Control: C57BL/6J	Age	3 M, 14 M		[197]
		Sex	Male		
		Number	3 M (3), 14 M (6–8)		
		Backcrosses	N/A		
*Lrrk2* ^p.G2019S^	C57BL/6-*Lrrk2^tm4.1Arte^* Control: C57BL/6NTac	Age	8–10 M		[198]
		Sex	Male		
		Number	WT (26), KI (22)		
		Backcrosses	N/A		
*Lrrk2* ^p.G2019S^	C57Bl/6J-*Lrrk2*^p.G2019S^ [175,309] Control: C57Bl/6J	Age	3 M, 6 M		[295]
		Sex	Male		
		Number	3		
		Backcrosses	N/A		
*Lrrk2* ^p.G2019S^	C57Bl/6J-*Lrrk2*^p.G2019S^ [309] Control: C57Bl/6J-*Lrrk2*	Age	4 M, 18 M		[204]
		Sex	Male & Female		
		Number	4		
		Backcrosses	N/A		
*Lrrk2* ^p.G2019S^	C57Bl/6J-*Lrrk2*^p.G2019S^ [309] Control: C57Bl/6J-*Lrrk2*	Age	4 M		[213]
		Sex	Male & Female		
		Number	4		
		Backcrosses	N/A		
*Lrrk2* ^p.G2019S^	C57Bl/6NTac-*Lrrk2*^p.G2019S^ Control: C57Bl/6NTac	Age	10–12 W		[205]
		Sex	Male		
		Number	4–5		
		Backcrosses	4		
*Lrrk2* ^p.G2019S^	C57Bl/6NTac-*Lrrk2*^p.G2019S^ Control: C57Bl/6NTac	Age	P21		[206]
		Sex	Male & Female		
		Number	WT (16), KI (20)		
		Backcrosses	N/A		
*Lrrk2* ^p.G2019S^ *Lrrk2* ^−/−^	B6.Cg-*Lrrk2^tm1.1Hlme^*/J (JAX 030961) https://www.jax.org/strain/030961 C57BL/6-*Lrrk2^tm1.1Mjff^*/J (JAX 016121) https://www.jax.org/strain/016121 Control: C57BL/6J	Age	4–6 M		[199]
		Sex	Male & Female		
		Number	WT (5), KI (14), KO (6)		
		Backcrosses	N/A		
*Lrrk2* ^−/−^	C57BL/6-*Lrrk2^tm1.1Mjff^*/J (JAX 016121) https://www.jax.org/strain/016121 Control: C57BL/6NJ (JAX 005304) https://www.jax.org/strain/005304	Age	8 W		[108]
		Sex	Male & Female		
		Number	8		
		Backcrosses	N/A		
*Lrrk2* ^−/−^	C57BL/6.129/Sv-*Lrrk2*^−/−^ [310] Control: C57BL/6	Age	2 M, 7 M, 24 M		[229]
		Sex	N/A		
		Number	3–4		
		Backcrosses	N/A		
*PRKN* ^p.Q^ ^311T^ ^er^	C57BL/6N-Tg(DAT-PRKN*Q311TERM) Control: C57BL/6N	Age	1 M, 6 M		[227]
		Sex	Male & Female		
		Number	10–11		
		Backcrosses	N/A		
*Prkn* ^p.R275W^	C57BL/6.C57BL/6NTac-*Prkn*^p.R275W^ Control: C57BL/6 (Charles River)	Age	1 M		[105]
		Sex	Male & Female		
		Number	3		
		Backcrosses	N > 10		
*Prkn* ^p.S65A^	C57BL/6J.C57BL/6NTac-*Prkn*^p.S65A^ Control: C57BL/6J	Age	12 M, 18 M		[104]
		Sex	Male/Female		
		Number	WT_12M_ (25), KI_12M_ (26), WT_18M_ (16), KI_18M_ (19)		
		Backcrosses	N/A		
*Pink1* ^p.G309D^	129/SvEv-*Pink1*^p.G309D^ Control: 129/SvEv	Age	16 M		[248]
		Sex	Male & Female		
		Number	WT (5), KO (6)		
		Backcrosses	N/A		
*Pink1* ^p.K219M^ *Pink1* ^p.G309D^	ADV-mediated *Pink1*^p.K219M^ injection on C57BL/6J;SJL ADV-mediated *Pink1*^p.G309D^ injection on C57BL/6J;SJL ADV-mediated WT *Pink1* injection on C57BL/6J Control: ADV-mediated GFP injection on C57BL/6J	Age	8–10 W		[290]
		Sex	Male		
		Number	3–4		
		Backcrosses	N/A		
*Pink1^−/−^*	C57BL/6N-*Pink1^−/−^* Control: C57BL/6N	Age	20 W, 40 W, 60 W		[247]
		Sex	Male & Female		
		Number	7–17		
		Backcrosses	N/A		
*Pink1^−/−^* (exons 1)	C57BL/6J;129/Sv-*Pink1^−/−^* Control: C57BL/6J;129/Sv	Age	N/A		[296]
		Sex	N/A		
		Number	7		
		Backcrosses	N/A		
*Pink1^−/−^* (exons 2–5)	C57BL/6;129/Sv-*Pink1^−/−^* Control: C57BL/6;129/Sv	Age	2–3 M, 8–9 M		[244]
		Sex	N/A		
		Number	2–3 M (4), 8–9 M (6–7)		
		Backcrosses	N/A		
*Pink1^−/−^* (exons 2–5)	C57BL/6J;129/SvEv^Brd^-*Pink1^−/−^* Control: C57BL/6J;129/SvEv^Brd^	Age	8 W		[245]
		Sex	N/A		
		Number	WT (2), KO (3)		
		Backcrosses	N/A		
*Pink1^−/−^* (exons 4–5)	C57BL/6;129/Sv-*Pink1^−/−^* Control: C57BL/6;129/Sv	Age	2 M, 6 M, 8.5 M, 12 M		[246]
		Sex	Male		
		Number	WT (5), KO (6)		
		Backcrosses	N/A		
*Pink1^−/−^* (exons 4–5)	C57BL/6.129/Sv-*Pink1^−/−^* [246] Control: C57BL/6	Age	7–8 M		[112]
		Sex	Male		
		Number	WT (14), KO (13)		
		Backcrosses	N > 15		
*Pink1^−/−^*	B6.129S4-*Pink1^tm1Shn^*/J (JAX 017946) https://www.jax.org/strain/017946 Control: C57BL/6J	Age	2 M, 3 M, 4 M, 5 M, 6 M		[249]
		Sex	Male		
		Number	18		
		Backcrosses	N/A		
*DJ-1* ^−/−^	B6.129-*Park7^tm1Mak^* [258] Control: C57BL/6	Age	10 M		[263]
		Sex	Male & Female		
		Number	5–7		
		Backcrosses	N > 7		
*DJ-1*^−/−^ (exons 1–5)	C57BL/6;129-*DJ-1*^−/−^ [311] Control: C57BL/6	Age	8–10 W	Suggested control: C57BL/6;129	[266]
		Sex	Male		
		Number	3		
		Backcrosses	N/A		
*DJ-1*^−/−^ (exons 1–5)	C57BL/6;129-*DJ-1*^−/−^ [311] Control: C57BL/6	Age	8–10 W	Suggested control: C57BL/6;129	[266]
		Sex	Male		
		Number	3		
		Backcrosses	N/A		
*DJ-1*^−/−^ (exon 2)	C57BL/6J;129/SvJ-*DJ-1*^−/−^ Control: C57BL/6J;129/SvJ	Age	2 M, 5 M, 14 M, 23 M		[257]
		Sex	Male		
		Number	12–16		
		Backcrosses	N/A		
*DJ-1*^+/−^ (exon 2)	B6.Cg-*DJ-1*^+/−^ B6.Cg-*Park7^tm1Shn^*/J (JAX 006577) https://www.jax.org/strain/006577 Control: C57BL/6	Age	8–10 W		[264]
		Sex	Male		
		Number	WT (5), KO (5)		
		Backcrosses	N/A		
*DJ-1*^−/−^ (exon 2)	B6;129-*DJ-1*^−/−^ [262] Control: B6;129	Age	8 W		[265]
		Sex	N/A		
		Number	4		
		Backcrosses	N/A		
*DJ-1*^−/−^ (exon 2)	B6;129-*DJ-1*^−/−^ [262] Control: B6;129	Age	4–8 W		[267]
		Sex	Male		
		Number	4		
		Backcrosses	N/A		

Abbreviations: W: x-week-old; M: x-month-old; Male & Female: mixed-sex mice; Male/Female: male and female mice analyzed separately; TG: Transgenic; KI: Knock-in; N/A: Not available; Number: number of animals used per group; Backcrosses: number of backcross generation. Note: All web resources URLs listed in this table were accessed on 30 April 2026.

**Table 6:** Examples of classical mouse behavioral assessments applied in PD research.

Classification	Behavioral Tests	PD-Related Symptoms	Applied in Mouse Models
Comprehensive	Open field test	Parkinsonian syndromes and Anxiety [313,314,315]	*SNCA*, *LRRK2*, *PRKN*, *PINK1*, and *DJ1*
Motor	Rotarod test	Parkinsonian syndromes [316,317,318,319]	*SNCA*, *LRRK2*, *PRKN*, *PINK1*, and *DJ1*
	Pole test	Bradykinesia [318,319,320,321,322]	*SNCA*, *LRRK2*, *PRKN*, *PINK1*, and *DJ1*
	Beam walking test	Postural instability [317,318,322,323,324]	*SNCA*, *LRRK2*, *PRKN*, *PINK1*, and *DJ1*
	Hanging test	Dystonia [81,318]	*SNCA*, *LRRK2*, *PRKN*, *PINK1*, and *DJ1*
	DigiGait test	Gait disturbance [325,326,327]	*SNCA*, *LRRK2*, *PRKN*, and *PINK1*
Non-motor	Electroencephalogram and EMG	Sleep and Circadian rhythms disorder [328,329]	*SNCA* and *LRRK2*
	Buried food-seeking test	Olfactory disorder [330,331,332]	*SNCA*, *LRRK2*, and *PINK1*,
	Voiding spot assay	Urinary dysfunction [333]	*SNCA*
	Metabolic cage assay	Constipation, GI and Urinary dysfunction [334]	*SNCA*, *PRKN*, and *DJ1*
	Whole gut transit time	GI [136]	*SNCA*
	Forced swim test	Depression [314,335]	*SNCA*, *LRRK2*, and *PRKN*
	Elevated maze test	Anxiety and fear [336]	*SNCA*, *LRRK2*, *PRKN*, *PINK1*, and *DJ1*
	T/Y maze test	Cognitive impairment [337,338,339]	*SNCA*, *LRRK2*, *PRKN*, and *PINK1*
	Barnes maze	Learning and Memory dysfunction and Bradykinesia [340]	*SNCA* and *PRKN*
	Morris water maze	Learning and Memory dysfunction [314,341]	*SNCA*, *LRRK2*, *PRKN*, and *PINK1*

**Table 7:** Variables contributing to experimental discrepancies in mouse models of PD.

Variable	Comment
Mouse strain used	Variation in genetic background and behavior
Genetic variants introduced	Different variants have differential effects
Transgenic technology used	Impacts expression levels, tissue distribution and cellular specificity of pathology
Age of the animals	Age-related differences in biological effects
Period of phenotypic examination	Required to assess progressive nature of the pathology
Protocols used for phenotyping	Different assessment methods measure different aspects of pathology, motor, non-motor and cognitive behavior.

## Data Availability

No new data were created or analyzed in this study. All information was obtained from publicly available literature.

## Abbreviations

The following abbreviations are used in this manuscript:

6-OHDA	6-Hydroxydopamine
AAV	Adeno-Associated Virus
ATP	Adenosine Triphosphate
ATP13A2	ATPase 13A2
BAC	Bacterial Artificial Chromosome
Bax	Bcl-2–Associated X Protein
Bcl-2	B-Cell Lymphoma 2
BIIB054	human-derived α-syn antibody
C57BL/6J	Inbred Laboratory Mouse Strain
CMV	Cytomegalovirus
CRISPR-Cas9	Genome Editing System
DaT-SPECT	Dopamine Transporter SPECT
DJ-1	Parkinsonism associated deglycase DJ-1
EMG	Electromyography
ER	Endoplasmic Reticulum
FVB/N	Inbred Laboratory Mouse Strain
GFAP	Glial Fibrillary Acidic Protein
GI	Gastrointestinal
IL	Interleukin
LRRK2	Leucine-Rich Repeat Kinase 2
MDS-UPDRS	Movement Disorder Society-United Parkinson’s Disease Rating Scale
MOM	Mitochondrial outer membrane
MPTP	1-Methyl-4-Phenyl-1,2,3,6-Tetrahydropyridine
mRNA	Messenger RNA
PAC	P1-Derived Artificial Chromosome
PARK	Parkinsonism-Associated Gene Loci
PCR	Polymerase chain reaction
PD	Parkinson’s Disease
PDGFB	Platelet-Derived Growth Factor Subunit B
PINK1	PTEN-Induced Kinase 1
qPCR	quantitative PCR
qRT-PCR	quantitative real-time PCR
SNARE	Soluble NSF Attachment Protein Receptor
SNpc	Substantia nigra pars compacta
SNP	Single nucleotide polymorphism
SYNJ1	Synaptojanin 1
TH	Tyrosine Hydroxylase
TNF	Tumor Necrosis Factor
